# Genetics and Distribution of the Italian Endemic *Campanula fragilis* Cirillo (Campanulaceae)

**DOI:** 10.3390/plants13223169

**Published:** 2024-11-11

**Authors:** Daniele De Luca, Emanuele Del Guacchio, Paola Cennamo, Francesco Minutillo, Liliana Bernardo, Paolo Caputo

**Affiliations:** 1 Department of Biology, University of Naples Federico II, 80139 Naples, Italy or daniele.deluca@unisob.na.it (D.D.L.); pacaputo@unina.it (P.C.); 2 Department of Humanities, University of Naples Suor Orsola Benincasa, 80132 Naples, Italy; paola.cennamo@unisob.na.it; 3 Botanical Garden of Naples, University of Naples Federico II, 80139 Naples, Italy; 4 Via Cuostile 3, 04024 Gaeta, Italy; framinutillo@libero.it; 5 Department of Biology, Ecology and Earth Sciences, University of Calabria, 87036 Rende, Italy; liliana.bernardo@unical.it

**Keywords:** DNA barcoding, haplotype diversity, maxent modeling, Mediterranean rupiculous flora, plant taxonomy, protected areas

## Abstract

*Campanula fragilis* Cirillo is a species distributed in central and southern Italy and includes two subspecies with uncertain taxonomic position and distribution. By means of nuclear and chloroplast markers, we attempted at testing the genetic distinctness of the two subspecies, as well as their possible correspondence with geographical or ecological patterns. After a revision of geographic occurrences based on herbarium data, we carried out species distribution modeling to assess the present and future distribution of this species under different ecological variables, also for conservation purposes. Our findings support the recognition of two weakly differentiated taxa, here accepted at subspecific rank, in agreement with the current taxonomic treatment. We found that *C. fragilis* subsp. *cavolinii* is monophyletic and limited to mountains and hills of central Italy. On the contrary, *C. fragilis* subsp. *fragilis* shows a higher genetic variability and a broader distribution in central and southern Italy, with a wider altitudinal range from coasts to mountain cliffs. We confirmed that both subspecies are narrowly calcicolous and have similar ecological requirements, but *C. fragilis* subsp. *cavolinii* occurs in colder habitats. Our results forecast a significant distribution contraction in the long term.

## 1. Introduction

The distribution patterns of biodiversity are the intricate results of the interaction of various factors (e.g., degree of isolation, climate, habitat patchiness, mating system, anthropogenic impact, and, more generally, abiotic and biotic interactions), which in turn cause non-random distribution of endemics, more frequently found in the so-called biodiversity hotspots [1]. With its unique geographical position in the Mediterranean basin hotspot, the Italian peninsula is itself a famous crucible for the production of diversity due to its climate patterns and its tectonic and paleoclimatic history [2,3]. Its prominent development along longitude generates a variety of different climates; in addition, the Apennine chain hinders both east-west distribution of low-altitude populations and, being highest in its central section, often prevents them from being distributed in the whole of mainland Italy [4,5]. For these reasons, southern and central Italy are home to a large number of endemic species and subspecies (e.g., [6,7,8]).

The genus *Campanula* L. (Campanulaceae) encompasses from a minimum of 350 to approximately 600 species [9] (449 according to [10]), of which about 250 occur in the Mediterranean area [11,12]. Italy includes 57 species (or 65 taxa, including subspecies), 21 of which are endemic [8]. Among the southern-central Italian endemics of the genus, *C. fragilis* Cirillo is a small isophyllous suffruticous chamaephyte [13]. It is naturally distributed from Lazio and Abruzzo, through Molise and Campania, to Basilicata and Calabria [8]. The ancient indications for Sicily [14] and Favignana island [15], as well as that for Dalmatia (ex Jugosalavia) [16] and Nice (southern France) [17], were never confirmed [18,19] and are nowadays neglected [8]. Further details about these ancient indications will be discussed in a separate paper. Similarly, the more recent reports from Veneto by D’Errico [20,21] are certainly erroneous [22], as well as those for Lombardy [23,24]. Being a very attractive plant (“pulcherrima planta” [25]), it has been cultivated for ornamental purposes since time [26,27,28,29] and naturalized in Liguria [30] and, outside Italy, in Great Britain and France [31,32], while it is only a casual alien in Germany [33] and Iceland [34].

From a phylogenetic point of view, *Campanula fragilis* belongs to the “Cam12” clade of the genus as recovered by Mansion et al. [9]. This clade is mainly composed of heterophyllous species (“harebells” by common name), together with a small number of isophyllous species, such as *C. elatinoides* Moretti, *C. isophylla* Moretti, and *C. fragilis* (cf. [35]). This latter species shows sufficient discontinuity in the variation pattern to have been divided into two subspecies [36], i.e., *C. fragilis* subsp. *fragilis*, occurring in the southern sector of the species range, and subsp. *cavolinii* (Ten.) Damboldt, limited to central Italy. While this treatment has been largely accepted (e.g., [8,13,37,38]), the exact distribution of the two subspecies is still uncertain due to the weakness of diagnostic features and extreme morphological variability. In fact, *C. fragilis* subsp. *cavolinii* was reported by Damboldt [36] only for the mounts of Abruzzo and for a single locality in Lazio (Subiaco), while the autonymous subspecies would be widespread from southern Lazio to southern Calabria. However, *C. fragilis* subsp. *cavolinii* has been reported in time for several southernmost localities (e.g., [39]), while according to [8], the autonym subspecies does not occur in Lazio, where its presence has been debated [36,37,40,41,42,43]. In addition, after the biochemical study by Frizzi and Tammaro [35], the genetic relationships between the two subspecies have never been thoroughly investigated.

It is to be noted that Castroviejo et al. [44] synonymized *C. fragilis* with *C. garganica* Ten., which would also be included in their treatment, for example, *C. poscharskyana* Degen and *C. fenestrellata* Feer, apparently without any supporting study. This synonymization is not only contrary to the opinion of most authors (e.g., [10,11,45]), based on evident and constant morphological differences (e.g., [13,36,37,46,47,48,49,50]), but it is definitively ruled out by karyological [36,41,51,52], biochemical [35], and molecular studies [9,53,54,55,56,57,58,59]. These studies pointed out a segregation of *C. fragilis* with *C. isophylla* in a different clade than *C. garganica* and its allied, mostly eastern species. Therefore, we will not give further attention to such taxonomic treatment.

In this study, we carried out a molecular analysis on fresh and herbarium samples of *C. fragilis* across its known distribution range to shed light on the genetic differentiation between the two subspecies. We also modeled their present and future potential distributions for conservation purposes.

## 2. Materials and Methods

### 2.1. Sample Collection and DNA Extraction

**Figure 1:**
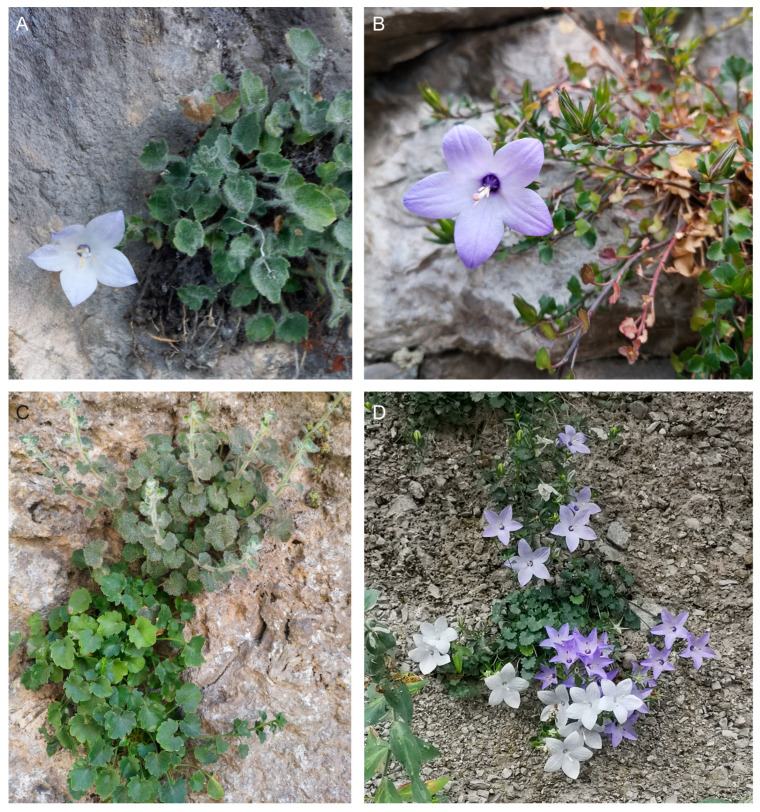
*Campanula fragilis* in its habitat. (**A**) *C. fragilis* subsp. *cavolinii* (Scanno, P. Caputo); (**B**) *C. fragilis* subsp. *fragilis* (Tramonti, D. De Luca); (**C**) coexistence of glabrous and tomentose individuals within the same population of *C. fragilis* subsp. *fragilis* (Tramonti, D. De Luca); (**D**) intra-individual variability of flower color in *C. fragilis* subsp. *fragilis* (Maiori, D. De Luca).

We collected leaf material from 43 individuals of *C. fragilis* across the distribution range (Table 1, Figure 1). The specimens were preliminarily identified following the keys and descriptions in Damboldt [36] and, in some cases, re-labeled. Ten specimens were identified as *C. fragilis* subsp. *cavolinii* (‘cavolinii’ in Table 1), and 33 as *C. fragilis* subsp. *fragilis* (‘fragilis’ in Table 1). It is to be noted that one individual (LAZ4), originally identified as *C. fragilis* subsp. *cavolinii* (RO, Herb. Anzalone), was re-identified by us as subsp. *fragilis*. Three samples of *C. isophylla* Moretti (ISO1-ISO3) were included in the analysis to verify the phylogenetic relationships of this species with *C. fragilis* since these species form an unresolved clade in the phylogeny [9]. Finally, *C. versicolor* Andrews subsp. *tenorei* (Moretti) I.Janković and D.Lakušić, endemic to Italy as well, were chosen as an outgroup based on previous phylogenies [9,60]. Two to three leaves were collected from each individual plant, indicated with a code in Table 1, and subjected to DNA extraction.

Total DNA was extracted using the NucleoSpin® Plant II kit (Macherey-Nagel, Düren, Germany) following the manufacturer’s protocol with buffer PL1. Plant material (around 25 mg) was ground to powder using Mixer Mill 300 (Retsch®, Verder Scientific, Haan, Germany). The quality and quantity of extracted DNA were evaluated by 0.8% gel electrophoresis using the high-molecular-weight marker HyperLadder™ 1Kb (Bioline, Meridian Bioscience, Cincinnati, OH, USA).

### 2.2. Marker Selection, Amplification, and Sequencing

To identify putative barcoding regions to distinguish *C. fragilis* subsp. *cavolinii* from subsp. *fragilis* and to assess their phylogenetic relationships, we tested several markers. After excluding the chloroplast regions *acc*D-*psa*I, *clp*P, and *trn*S^GCU^-*trn*G^GCC^ [61] and the nuclear region ETS [62], that resulted in a lack of or non-target amplification, we amplified six molecular markers, one nuclear (ITS), and five chloroplast intergenic spacers (*ndh*J-*trn*F, *pet*B-*pet*D, *pet*N-*psb*M, *trn*H-*psb*A, and *trn*F-*trn*L). Polymerase chain reactions (PCRs) were carried out into a final volume of 20 µL each containing 7–10 ng DNA, 2X Phire™ Plant Direct PCR Master Mix (Thermo Fisher Scientific, Waltham, MA, USA), 250 nM forward and reverse primers, and water to volume. PCR conditions and primer references [63,64,65,66,67,68,69] are listed in Table S3. The success of amplification was determined by electrophoresis in 1.5% agarose gel stained with SafeView^™^ Classic (ABM®, Richmond, BC, Canada) running in 0.5X TBE buffer; the gel was visualized under UV using the TFX-20.M transilluminator (Vilber Lourmat, Marne-la-Vallée, France). The amplified products were purified using 20% PEG (Polyethylene Glycol) 8000 (AppliChem, Darmstadt, Germany) and quantified on a 1.5% agarose gel with the 100bp Opti-DNA Marker (ABM®, Richmond, BC, Canada). Sequencing reactions were carried out in a final volume of 5 µL using the BrightDye® Terminator Cycle Sequencing Kit (MCLAB, Harbor Way, San Francisco, CA, USA) and purified using the BigDye® Xterminator^™^ Purification Kit (Applied Biosystems, Thermo Fisher Scientific, Foster City, CA, USA). Capillary electrophoresis was carried out in the Applied Biosystems® 3130 Genetic Analyzer (Applied Biosystems, Thermo Fisher Scientific, Foster City, CA, USA).

### 2.3. Sequence Analysis, Haplotype Identification, and Phylogenetic Inference

Electropherograms of ITS sequences were visually inspected for ambiguities; all the sequences were aligned using the ClustalW algorithm [70] as implemented in the BioEdit v7.2.6 software [71]. Standard IUPAC ambiguity codes were used for ITS when base peaks overlapped or the lower peak was at least one-third in height, which was the highest one. All sequences were deposited in DDBJ (see Data availability section for details). Plastid haplotypes for *C. fragilis* were inferred in mothur v1.40.5 [72] using the function *unique.seqs*, which is not affected by the occurrence of missing loci in the concatenated matrix. The resulting haplotypes were plotted on a map (“Esri.WorldShadedRelief”) using the R [73] packages *ggplot2* [74] and *leaflet* [75]. The ITS alignment was visually inspected for the presence of character states discriminative of the two subspecies.

The best-fitting evolution model was computed for each marker in jModelTest v2.1.3 [76] using the corrected Akaike information criterion [77]. An incongruence length difference (ILD) test was carried out in Nona v2.0 [78] within Winclada v1.00.08 [79] to test the putative incongruence of nuclear and chloroplast partitions prior to concatenation; default values were used for the analysis. Bayesian phylogenetic trees were inferred for the nuclear, chloroplast, and concatenated datasets in MrBayes 3.2 [80] under the substitution models resulted from the jModelTest analysis and running two replica runs and four chains for 1,000,000 generations and sampling chains every 1000 steps. The resulting trees were graphically edited in TreeGraph 2 v2.15.0-887 [81], and nodes with p.p. < 0.9 were collapsed.

### 2.4. Species Distribution Modeling

We used the maximum entropy modeling algorithm implemented in the software MaxEnt v3.4.4 [82] to predict the present and future geographic distribution of the two subspecies of *C. fragilis* under different climatic scenarios. This algorithm uses environmental variables and occurrence data to estimate a target probability distribution that represents the suitability of conditions for the species under investigation. Distribution records for both subspecies were collected from GBIF (https://www.gbif.org/; accessed on 7 March 2024) after deleting records referring to plants cultivated, vaguely localized (e.g., Kingdom of Naples), erroneously identified, or not accompanied by an image. The database was integrated with the occurrence data of the present study and of further specimens from APP and CLU (see Table S2). Nineteen bioclimatic variables with a 2.5’ resolution for the present (1970–2000), mid-term (2040–2060), and long-term (2080–2100) periods under four shared socioeconomic pathways (SSPs: SSP126, SSP245, SSP370, and SSP585) were downloaded from WorldClim v2.1 (http://www.worldclim.org/; accessed on 8 March 2024). The variables were extracted at occurrence points using the R [73] package *raster* [83] and tested for correlation (Pearson’s) among them using the package *dismo* [84]. Highly correlated variables (|r| ≥ 0.8) were removed in order to reduce the impact of collinearity in maxent modeling. The MaxEnt analysis was carried out by running the entropy model ten times with random seeds and a bootstrap strategy. For each model, the distribution data were randomly split into two subsets: 75% as training data and the remaining 25% as testing data; the latter is used by the program to compute several statistics that are index of good fit of the model, as AUC and omission rate. To build reliable species distribution models, 10,000 pseudo-absence coordinates were randomly generated [85]. The area under the receiver operating curve (AUC) was used to evaluate the performance of the model [86]; AUC values > 0.8 (or close to 1), with the testing line (in blue) close to the training line (in red), indicate a good fit of the model to the given data. In addition, the minimum difference between training and testing AUC data (AUC Diff), where small differences are indicative of less overfitting present in the model, was considered [87,88]. Predicted habitats in ASCII format generated by MaxEnt analysis were imported in raster and plotted as four classes of potential habitats using *mapIT* [89] and *rgdal* [90]. The habitat classes were grouped according to their probability as follows: unsuitable (≤0.10), low potential (0.11–0.30), moderate potential (0.31–0.70), and high potential (≥0.71) [91].

### 2.5. Mapping of Occurrence Records on the Geo-Lithological and Protected Areas Layers

In order to verify the preference of *C. fragilis s.l.* for calcareous substrates, we plotted the 132 geo-reference records used for the MaxEnt model in the software QGIS v3.38.2-Grenoble (http://www.qgis.org; accessed on 25 August 2024) using the layer of the geo-lithological map of Italy available at Geoportale Nazionale Italiano (http://wms.pcn.minambiente.it/ogc?map=/ms_ogc/WMS_v1.3/Vettoriali/Carta_geolitologica.map; accessed on 25 August 2024). The same procedure was followed to plot the occurrences on the map of Italian protected areas (http://wms.pcn.minambiente.it/ogc?map=/ms_ogc/WMS_v1.3/Vettoriali/EUAP.map; accessed on 25 August 2024).

## 3. Results

### 3.1. Dataset Characteristics and Phylogenetic Inference

The nuclear alignment (Data S1) was composed of 868 characters (ITS1-5.8S-ITS2-28S), while the chloroplast one, resulting from the concatenation of five chloroplast regions (Data S2), was composed of 2637 characters (*ndh*J-*trn*F: 564 bp; *pet*B-*pet*D: 796 bp; *pet*N-*psb*M: 591 bp; *trn*H-*psb*A: 334 bp; *trn*F-*trn*L: 352 bp). Amplification was not successful in some individuals for the following markers: *ndh*J-*trn*F (BAS2), *pet*N-*psb*M (LAZ2), and *trn*F-*trn*L (ABR1 and *C. versicolor* samples). Furthermore, ITS sequences of BAS2 and *C. versicolor* were shorter than the others.

The best-fitting nucleotide evolutionary models computed for each marker were F81 (*pet*N-*psb*M), GTR (*ndh*J-*trn*F and *pet*B-*pet*D), HKY (*trn*H-*psb*A), and SYM+I (ITS). According to the ILD test, the intergenic spacer *ndh*J-*trn*F was found to be incongruent with the nuclear ITS dataset (*p* < 0.05) and was therefore excluded from the phylogenetic inference using the concatenated nuclear and chloroplast dataset. The resulting concatenated matrix was 2941 bp long and constituted, in order, by the following markers: *pet*B-*pet*D, *pet*N-*psb*M, *trn*H-*psb*A, *trn*L-*trn*F, and ITS (Data S3).

The consensus tree obtained from the Bayesian analysis of the nuclear dataset (Figure S1), rooted at *C. versicolor*, showed that *C. isophylla* is sister to *C. fragilis*. All specimens of *C. fragilis s.l.* formed a monophyletic group (p.p. = 1). Within this clade, all *C. fragilis* subsp. *cavolinii* samples (hereafter ‘cavolinii’) formed a highly supported clade (p.p. = 1); on the contrary, relationships between *C. fragilis* subsp. *fragilis* samples (hereafter ‘fragilis’) remained mostly unresolved. The only supported clade (p.p. = 0.97) included the ‘fragilis’ samples from the Cilento region (Mts. Alburni and Caggiano) and one sample from Calabria (CAL3). The individuals collected from the population of Mt. Orlando (LAZ4-LAZ7), of debated identification, were collapsed as the other ‘fragilis.’

The Bayesian chloroplast tree (Figure S2) confirmed the sister relationship of *C. isophylla* to *C. fragilis*. Within the *C. fragilis s.l.* clade, no monophyly was detected either for the samples of *C. fragilis* subsp. *cavolinii* or for those of *C. fragilis* subsp. *fragilis*. However, several small groups coherent with geography were observed. Among the ‘cavolinii’ specimens, those from Abruzzo (ABR1-ABR4), those from Molise (MOL1-MOL3), and two from close localities in Lazio (LAZ1-LAZ2) were grouped, respectively, in three different clades, all with p.p. = 1. Only the sample from Trisulti (LAZ3) was not included in any of these clades. For *C. fragilis* subsp. *fragilis*, we found the following highly supported (p.p. = 1) groups: a first one formed by samples from the Tyrrhenian section of Basilicata and north Calabria (BAS1, CAL1, CAL3, CAL4, and CAL8); a second one with samples from the easternmost sector of the range (BAS3, CAL6, and CAL7) plus another one from the Tyrrhenian side (CAL2); a third one with samples from the island of Capri (CAM5-CAM7); and a fourth one with two samples from Mts. Alburni (CAM13-CAM14). The relationships among the other specimens were not resolved.

**Figure 2:**
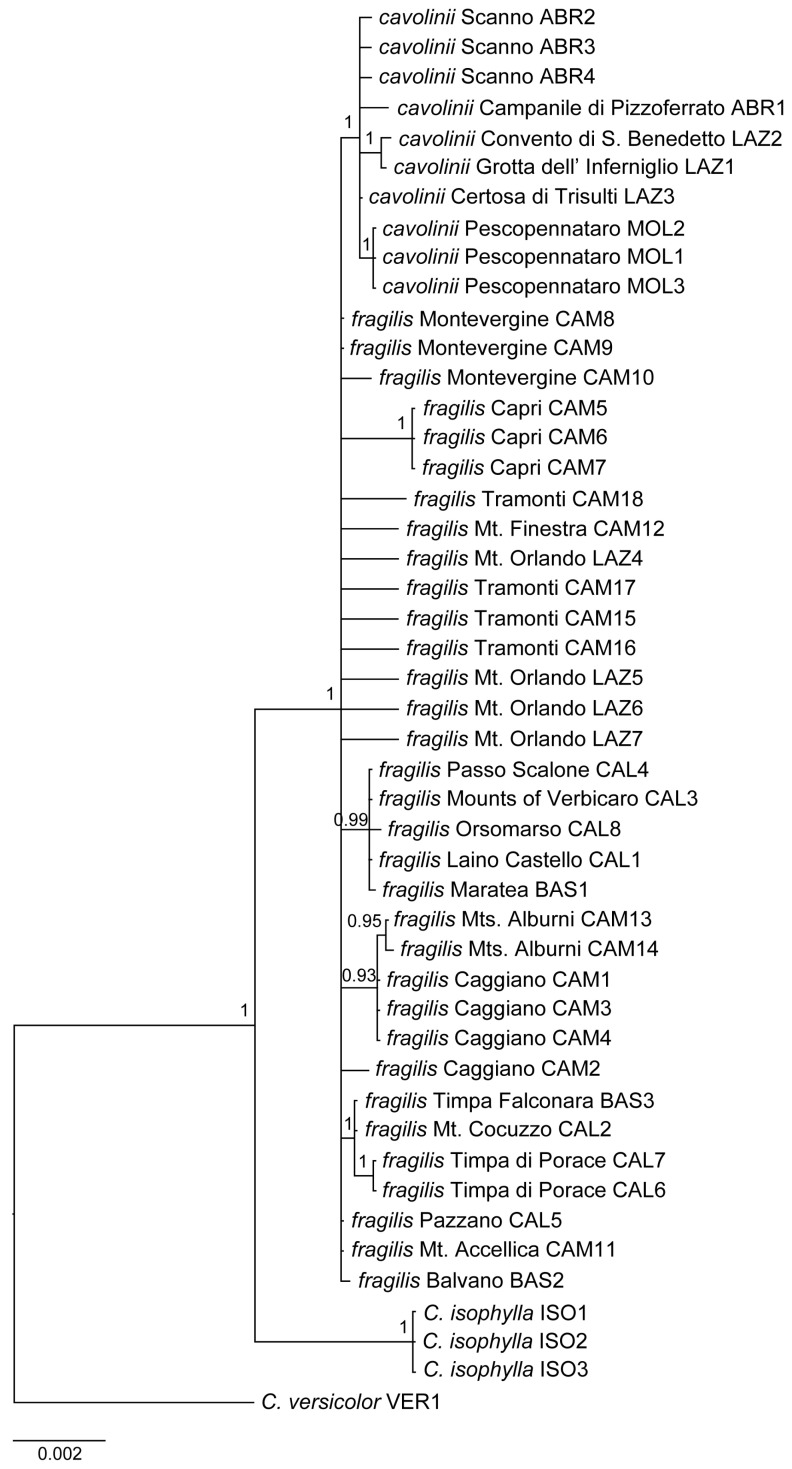
Bayesian consensus tree based on the concatenated (nuclear and chloroplast) matrix. Numbers at nodes indicate posterior probabilities.

The Bayesian consensus tree of the merged dataset without *ndh*J-*trn*F (Figure 2), again rooted with *C. versicolor*, confirmed that *C. isophylla* is sister to *C. fragilis*, which in turn forms a monophyletic unit (p.p. = 1). Within this clade, all ‘cavolinii’ samples were included in a highly supported clade (p.p. = 1). On the contrary, ‘fragilis’ individuals did not form a monophyletic unit; however, several specimens of this subspecies grouped together with high support (p.p. > 0.9). The first monophyletic group (p.p. = 1) included samples from Pollino Massif (BAS3, CAL6, and CAL7) and another individual from the Calabrian Apennines (CAL2). A second group (p.p. = 0.93) included the samples from the Mts. Alburni and from Caggiano (CAM13 and CAM14, and CAM1, CAM3, and CAM4, respectively). A third monophyletic unit (p.p. = 0.99) was composed of specimens from Basilicata and northern Calabria (BAS1, CAL1, CAL3, CAL4, and CAL8). A fourth group (p.p. = 1) was represented by all the specimens from Capri.

### 3.2. DNA Barcoding and Haplotype Diversity

**Figure 3:**
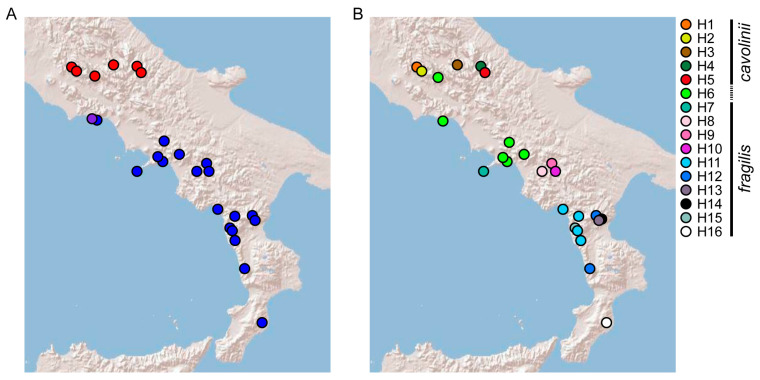
Genetic patterns of the sampled populations of *C. fragilis*. (**A**) nuclear signal: distribution of DNA barcodes (red = ‘cavolinii’ barcodes, blue = ‘fragilis’ barcodes, violet = intermediate barcodes); (**B**) plastid signal: haplotype distribution.

For DNA barcoding purposes, we found discriminating positions in the ITS1 and 5.8S regions. In our alignment (Data S1), positions 20 and 38 in the ITS1 showed states “G” and “C” in all ‘cavolinii’ samples and states “A” and “G” in all ‘fragilis’ specimens, respectively; at the position 311 (5.8S region), all the ‘cavolinii’ specimens presented a “T”, while the ‘fragilis’ samples a “C”. In these positions, the debated samples from Mt. Orlando showed ambiguities or were resolved as ‘fragilis’. Namely, LAZ4 and LAZ5 showed ambiguities in the three barcoding positions (“R”, “S”, and “Y”, respectively), while LAZ3 was resolved as ‘fragilis’ for the first two positions and showed an ambiguity (“Y”) only in the third one; finally, LAZ1 presented the character states of ‘fragilis’ in all the three barcoding positions. The ribosomal cistron was also the most variable region within each population sample. A graphical representation of the genetic makeup of *C. fragilis s.l.* based on nuclear DNA barcoding sites is provided in Figure 3A.

The concatenation of chloroplast markers resulted in 16 haplotypes (Table S3 and Data S4), of which 5 were unique to ‘cavolinii’ samples and 10 to ‘fragilis’ samples (Figure 3B). Only one haplotype (H6), the most widespread, was shared both by groups because it occurred in the northernmost samples of ‘fragilis’ in Campania (CAM8-CAM12, CAM15-CAM18) and Lazio (LAZ4-LAZ7), as well as in a ‘cavolinii’ individual (LAZ3). Therefore, this H6 haplotype occurred in a geographical intermediate position between the two groups. The second most widespread haplotype (H11) occurred in some specimens from the Tyrrhenian side of Basilicata and Calabria (BAS1, CAL1, CAL3, and CAL4). All the other chloroplast haplotypes were typical of a particular individual or sampling location.

### 3.3. Potential Habitat Suitability of C. fragilis Under Present and Future Climate Conditions

A total of 132 geo-referenced occurrence records were utilized to build the MaxEnt models (Table S2). Of these, 46 records were from unpublished herbarium data or personal observations (8 for ‘cavolinii’, and 38 for ‘fragilis’), plus 86 from GBIF (https://doi.org/10.15468/dl.9jvuur for ‘cavolinii’, i.e., 39 filtered, georeferenced records, and https://doi.org/10.15468/dl.vstg7f, i.e., 47 georeferenced records, for ‘fragilis’, accessed on 16 July 2024). Regarding the bioclimatic variables, after Pearson’s correlation analysis (Figure S3), 10 out of 19 were recovered as uncorrelated: Bio1 (annual mean temperature), Bio2 (mean diurnal range), Bio3 (isothermality), Bio6 (min temperature of coldest month), Bio7 (temperature annual range, i.e., Bio5-Bio6), Bio8 (mean temperature of wettest quarter), Bio12 (annual precipitation), Bio14 (precipitation of driest month), Bio15 (precipitation seasonality), and Bio19 (precipitation of coldest quarter); these variables were used for species distribution modeling in addition to the geo-lithological map. The inferred models showed high levels of predictive performance for both subspecies (Figure S4), with the following AUC values (±SD) for *C. fragilis* subsp. *cavolinii* and *C. fragilis* subsp. *fragilis*, respectively: AUCtraining = 0.983 and 0.968; AUCtest = 0.983 ± 0.007 and 0.953 ± 0.017; and AUCDiff = 0.001 and 0.015. Regarding ‘cavolinii’, the bioclimatic variable with the highest contribution to the model when used in isolation was precipitation seasonality (Bio15: 53.9%), followed by precipitation of the driest month (Bio14: 19.7%); all the other variables had a contribution lower than 6%. The bioclimatic variables that contained the most information that was not present in the others were precipitation of the driest month (Bio14: permutation importance, p.i., of 70.2%) and precipitation seasonality (Bio15: p.i. = 8.7%). The variable Bio15 was the most important for ‘fragilis’ as well (contribution of 33.1%), despite the fact that other variables had a relevant contribution to the distribution model, such as temperature annual range (Bio7: 17.2%), precipitation of the driest month (Bio14: 15.5%), and precipitation of the coldest quarter (Bio19: 10.1%). The geo-lithological layer contributed 15.3%. Exclusive information was found in the following variables: annual precipitation (Bio12: p.i. = 27.2%), precipitation of driest month (Bio14: p.i. = 25.6%), temperature annual range (Bio7: p.i. = 10.6%), as well as in the geo-lithological layer (p.i. = 13.1%).

**Figure 4:**
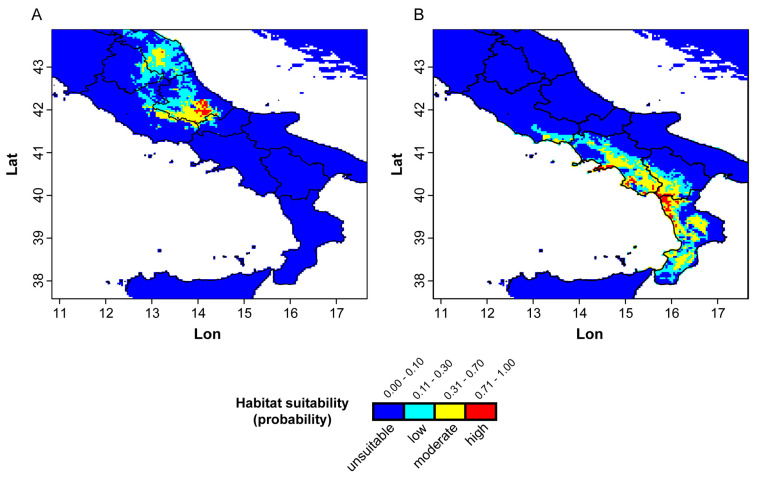
MaxEnt model of the predicted distribution of *C. fragilis* under current environmental conditions. (**A**) *C. fragilis* subsp. *cavolinii*; (**B**) *C. fragilis* subsp. *fragilis*.

The response curves of the ten selected bioclimatic variables to habitat suitability of ‘cavolinii’ and ‘fragilis’ are shown in Figure S5. Considering moderate to high probabilities of occurrence using relevant variables related to temperature, values of the mean annual temperature range (Bio1) for ‘cavolinii’ and ‘fragilis’ were 0–12 °C and 6–22 °C, respectively, and values of the minimum temperature of the coldest month (Bio6) from −10 to 0.5 °C and 0–13 °C; for the variables Bio7 and Bio8, no relevant differences were observed between the two subspecies, despite ‘cavolinii’ also occurring at lower temperatures. Regarding the variables related to precipitation, moderate to high probabilities of occurrence for annual precipitation (Bio12), precipitation of driest month (Bio14), and precipitation of coldest quarter (Bio19) were, in the order: ~600–1000 mm, 42–62 mm, and 130–215 mm for ‘cavolinii’ and ~700–1400 mm, 13–30 mm, and 230–520 mm for ‘fragilis’. The potential distribution of the two subspecies according to these variables is shown in Figure 4.

**Figure 5:**
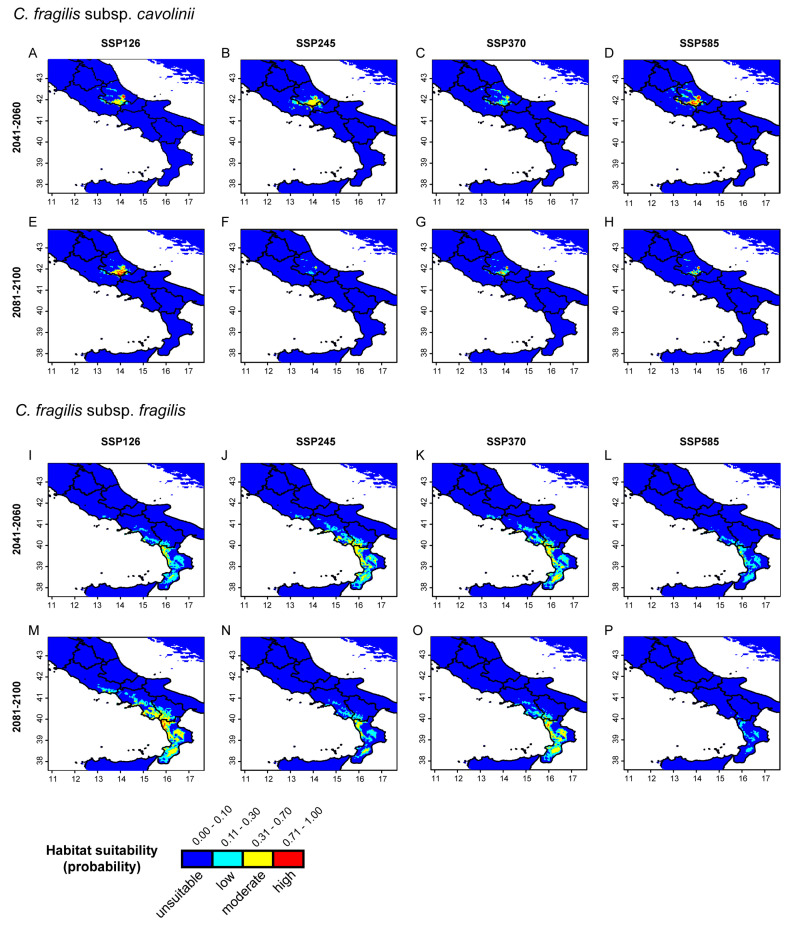
Maxent models of the predicted distribution of *C. fragilis s.l.* under future climatic conditions. (**A**) SSP126, (**B**) SSP245, (**C**) SSP370, and (**D**) SSP585 predicted scenarios for *C. fragilis* subsp. *cavolinii* during 2041–2060; (**E**) SSP126, (**F**) SSP245, (**G**) SSP370, and (**H**) SSP585 predicted scenarios for the same subspecies during 2081–2100; (**I**) SSP126, (**J**) SSP245, (**K**) SSP370, and (**L**) SSP585 predicted scenarios for *C. fragilis* subsp. *fragilis* during 2041–2060; (**M**) SSP126, (**N**) SSP245, (**O**) SSP370, and (**P**) SSP585 predicted scenarios for the same subspecies during 2081–2100.

The projected distribution of *C. fragilis s.l.* in the mid- (2041–2060) and long-term (2081–2100) periods was remarkably different for the two subspecies. For *C. fragilis* subsp. *cavolinii*, moderate to high probabilities of occurrence are reduced to the central–southwestern areas of the Abruzzo region (Figure 5A–D). In the two mid-term scenarios, SSP126 (Figure 5A) and SSP245 (Figure 5B), the distribution was comparable; in scenario SSP370, areas at moderate to high probabilities of occurrence were drastically reduced (Figure 5C), while in the climatic scenario SSP585, with the highest radiative force (8.5 W/m^2)^, such areas were even more extended compared to previous scenarios (Figure 5D). In long-term scenarios, *C. fragilis* subsp. *cavolinii* was still widely distributed under the SSP126 model (Figure 5E), but suitability areas remarkably decreased under the SSP245 model (Figure 5F), to slightly increase again under the models SSP370 (Figure 5G) and SSP585 (Figure 5H). For *C. fragilis* subsp. *fragilis*, moderate to high probabilities of occurrence were observed in the Sorrento Peninsula, southwestern Campania, Basilicata, and the western side of Calabria for mid-term SSP126 (Figure 5I), SSP 245 (Figure 5J), and SSP370 (Figure 5K) scenarios; in the SSP585 scenario (Figure 5L), only localities in the Sorrento peninsula showed the highest probability of occurrence, while moderate probabilities were limited to the north-western coastal localities of Calabria. In two long-term scenarios, specifically SSP126 and SSP370 (Figure 5M,O), the distribution of *C. fragilis* subsp. *fragilis* was comparable to present days and included, at least for scenario SSP126, the southern Lazio localities appearing in other mid-term scenarios. From model SSP245 (Figure 5N) to SSP585 (Figure 5P), we observed a progressive reduction in the potential distribution range, with only a few suitable areas in Calabria with moderate probability of occurrence and many low-potential habitats.

### 3.4. Substrate Preferences and Conservation of C. fragilis

**Figure 6:**
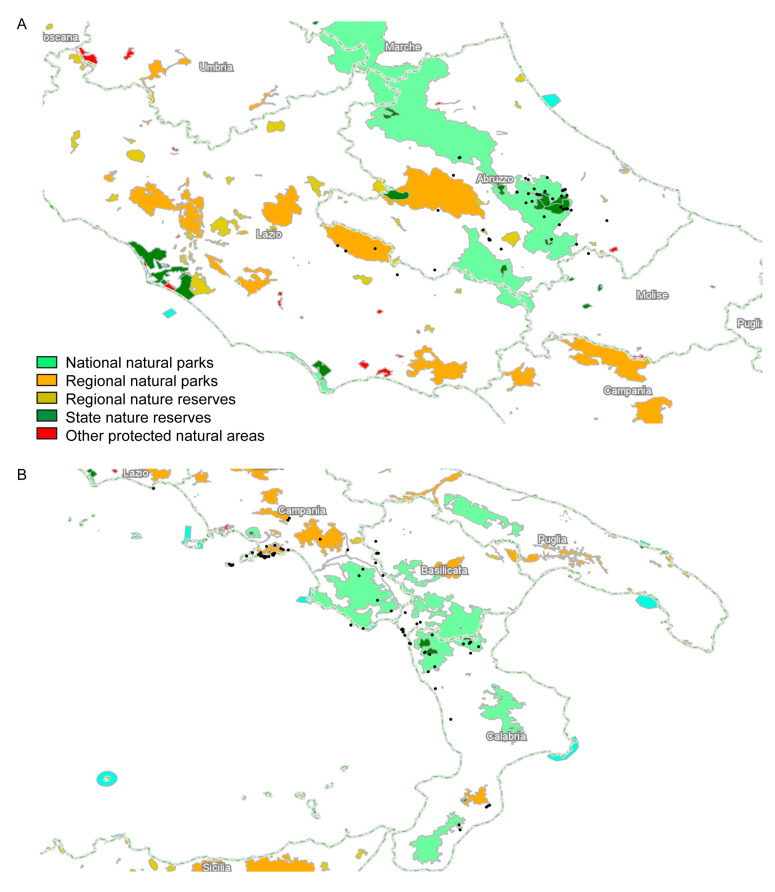
Distribution of geo-referenced occurrence records of *C. fragilis* within protected areas. (**A**) *C. fragilis* subsp. *cavolinii*; (**B**) *C. fragilis* subsp. *fragilis*.

The plotting of the abovementioned 132 occurrence records of *C. fragilis* on the geo-lithological map of Italy indicated that both subspecies occur on calcareous substrates (Figure S6). Limestones and dolomites constituted the predominant substrates for both subspecies; among the other substrates, *C. fragilis* subsp. *cavolinii* also occurred on calcareous marls (in the Abruzzo region), while both subspecies on deposits mostly made of flysch. Regarding the conservation of *C. fragilis*, most of the geo-referenced occurrence records fell within protected areas (Figure 6). For ‘cavolinii’, these areas correspond to the regional natural park of Mounts Simbruini in Lazio, the Gran Sasso and Monti della Laga National Park, the Maiella National Park, the Fara San Martino Palombaro and Valle dell’Orfento natural reserves, and the Gole del Sagittario regional reserve in Abruzzo (Figure 6A). For ‘fragilis’ (Figure 6B), in the Campania region, most of the occurrences were in the Mounts Lattari regional park (Sorrento peninsula), followed by the Mounts Picentini and Partenio regional parks, the Foce Sele-Tanagro natural reserve, and the Cilento and Vallo di Diano National Park; in Basilicata, all the points were outside protected areas; in Calabria, in the Pollino and Aspromonte National Parks, in the Valle del Fiume Argentino natural reserve, as well as in non-protected areas.

## 4. Discussion

This study provides robust support for distinguishing *C. fragilis* from the similar *C. isophylla* from a molecular standpoint. In fact, despite their taxonomic distinctness, they have never been put in doubt due to the constancy of diagnostic morphological characters and separate ranges [50]. From a molecular point of view, the two species are not resolved [9,12,54] or not completely resolved [56] in previous phylogenetic trees. We fully separated *Campanula isophylla* and *C. fragilis* in our nuclear, chloroplast, and concatenated Bayesian analyses based on a substantial number of individuals and informative characters. *Campanula isophylla*, a narrow endemic to western Liguria (N-W Italy), shows not only morphological resemblance to *C. fragilis* but also a remarkable similarity in ecology, growing exclusively on maritime limestone cliffs.

The evidence reported in the previous references and in other recent contributions [58] rules out any attempt to group together *C. isophylla* and *C. fragilis* with other isophyllous species of the amphi-Adriatic aggregate of *C. portenschlagiana* (cf. [44]). Similarly to what is found in the so-called “Palaeomediterraneae series” of *Cynanchica* P. Caputo & Del Guacchio, a mere morphological similarity between a western and an eastern Mediterranean group of species may not reflect closer phylogenetic relationships [92], and also in that case the two groups evolved independently. The clade composed of *C. fragilis* and *C. isophylla* is sister to a group including *C. rotundifolia* L., *C. scheuchzeri* Vill., and *C. pollinensis* Podlech, and this inclusive group is in turn sister to *C. elatines* L. and *C. elatinoides* [58]. All the mentioned relatives of the *C. fragilis*-*C. isophylla* clade are mountain species, with *C. rotundifolia* widespread in temperate to cold-temperate environments of Eurasia [10] but occurring in Italy exclusively in the Alps and Apennines [8]. For this reason, we may hypothesize that *C. fragilis* and *C. isophylla* share a Tyrrhenian coastal common ancestor, which reached isolation on maritime limestone cliffs from a widely distributed, cold-adapted species in an interglacial period.

At the infraspecific level, on the one hand, our results did not provide a clear separation of *C. fragilis* into two different clades corresponding to its two subspecies; on the other hand, however, a well-defined group composed of *C. fragilis* subsp. *cavolinii* is clearly outlined both in the nuclear tree and in the concatenated one. Despite this, the chloroplast tree is largely unresolved. Only an SW Tyrrhenian clade represented by five specimens originated in the Lucanian Apennines (Basilicata and northern Calabria), and a smaller group, including specimens from S-E Calabria and its Catena Costiera (Mt. Cocuzzo), are visible. However, these clades in the chloroplast tree (and, as a consequence, in the concatenated one) appear more related to geography and seed dispersal than to lineage sorting, as already known for other species (e.g., [93,94,95]). From an operational point of view, we demonstrated that the ITS marker is a useful tool for DNA barcoding up to the subspecies level. In addition, it can sometimes distinguish single population samples (e.g., Maratea, Orsomarso, Pizzoferrato, Timpa di Porace).

To explain the origin of *C. fragilis* subsp. *cavolinii* and its low genetic variability, Frizzi and Tammaro [35] did not exclude a founder effect from *C. fragilis* subsp. *fragilis*. However, in our opinion, the scattered but relatively wide occurrence of *C. fragilis* subsp. *cavolinii*, its different ecology (see above), and its haplotype variability (with one haplotype shared with the other subspecies in an intermediate area) seem rather suggestive of a peripatric speciation process. In our concatenated phylogenetic tree, monophyly is absent in *C. fragilis* subsp. *fragilis*, and the unresolved position of some samples with respect to the monophyletic subps. *cavolinii* could be interpreted as the result of an ancestral condition. In this scenario, the *C. fragilis* subsp. *cavolinii* could be derived from subsp. *fragilis* after the progressive divergence between northern and southern populations, in accordance with Frizzi and Tammaro [35] and as already hypothesized by Béguinot [96]. Of course, the topology of the obtained tree could also be due to the incapability of our markers to detect deep phylogenetic signals. A correlation between the molecular and morphological features of the two subspecies is beyond the scope of the present contribution. However, we found that the most reliable characteristic to distinguish between the two subspecies is the shape of calyx lobes (strictly lanceolate-linear and distantly separate in *C. fragilis* subsp. *cavolinii* vs. narrowly triangular and convergent at the base in *C. fragilis* subsp. *fragilis*); this character is better visible at anthesis. In addition, subsp. *cavolinii* often has a smaller corolla (cf. [36]), not much wider than the calyx, while the corolla is typically almost twice as long as the calyx lobes in subsp. *fragilis*. Further presumed diagnostic characters, such as the leaf shape and its ratio with the petiole, the length of hairs on stamen filaments (up to 1\2 of the width of the filament in *C. cavolinii* vs. c. 1/7 in *C. fragilis*) [36], the color of the corolla (paler in *C. cavolinii*) [48], or the coating of the hypanthium (farinose in *C. cavolinii*) [46], resulted unreliable or subjective and in some cases difficult to observe. Finally, it is to be noted that both subspecies show, often within the same population, densely grayish tomentose plants together with glabrous individuals. This occurs, for example, in the populations of Scanno for *C. fragilis* subsp. *cavolinii* and in those of Tramonti (Figure 1C) and Caggiano for *C. fragilis* subsp. *fragilis*, so denying any taxonomic value to this character (cf. [46,97]). As further proof, we found that hirsute or glabrous individuals within the same population resulted in genetically undistinguishable; for example, the hirsute CAM1-CAM2\CAM17-CAM18 vs. the glabrous CAM3-CAM4\CAM15-CAM16 for the populations from Caggiano and Tramonti, respectively. Accordingly, the specimens from southern Lazio can be attributed to the autonym subspecies despite being somehow intermediate from both the molecular and the morphological standpoints, as testified by different identifications over time (e.g., [8,42,47,98,99]). In the barcoding region of ITS1-5.8S, one specimen from this area (LAZ1) presented the character states of subp. *fragilis*, while the others (LAZ3, LAZ4, and LAZ5) shared ambiguous bases. The chloroplast haplotype (H6) of the same population was found just northward among ‘cavolinii’ populations and southward among ‘fragilis’ populations. These data confirm an admixture between the two subspecies. In conclusion, populations referable to the autonym subspecies occur from southern Lazio to Calabria, especially along the Tyrrhenian coast but also reaching the internal massifs and the Ionian side (Lazio, Campania, Basilicata, Calabria). *Campanula fragilis* subsp. *cavolinii* is endemic to the mountains of central Apennines (Abruzzo, Molise, Lazio). Sometimes, populations with intermediate features can be found outside southern Lazio. For example, some populations from Cilento show linear calyx lobes. Otherwise, Damboldt [36] himself listed the localities of *C. fragilis* subsp. *fragilis* and reported “Vallepietra” (Frosinone, Lazio), where *C. fragilis* subsp. *cavolinii* indeed occurs; this should suggest that the diagnostic features are often weak. Finally, at the present state of knowledge, the species is to be excluded from Sicily, Veneto, and Dalmatia.

The potential distribution range, indicated by the MaxEnt model (Figure 4), largely concurs, with some exceptions, with the known distribution of the species. Regarding *C. fragilis* subsp. *cavolinii*, besides the known distribution in Abruzzo, Lazio, and Molise, the model also predicted a moderate probability of occurrence northward in the Marche and Umbria regions, where it has never been reported [8]. On the contrary, the predicted distribution of the autonym subspecies completely matched the known occurrence records. The plotting of geo-referenced occurrence records on the geo-lithological map (Figure S6) confirmed that *C. fragilis* prefers calcareous cliffs [35,100,101]. However, studies on adaptation to carbonates would be desired. Thus, volcanic areas such as Ischia (Gulf of Naples, Campania) or Pontine Islands (Latina province, Lazio) are excluded, and effectively *C. fragilis* does not occur there. One might infer that this distribution may reflect instead different geological ages, as hypothesized by Del Guacchio and Caputo [102] for *C. imperati* Ten. and *C. suaveolens* Bertol. However, in our case, anciently emerged granitic massifs (e.g., Mt. Sacro in Cilento, Serre Massif in Calabria), fully included in the native range of *C. fragilis*, do not host it. Incidentally, we note that the indication for the area of the Roccamonfina Volcano (northern Campania) [103] is not confirmed and might be erroneous (A. Croce, pers. comm.). Finally, we also note that these results make very unlikely the ancient indication by Rafinesque [14] for Mt. Etna (Sicily).

The species can be found at sea level up to 1400 m a.s.l. (EDG and LB, pers. obs.), so it is relatively widespread along the altitudinal range. This adaptability, shared with other sympatric chasmophytes (e.g., *Brassica incana* Ten., *Lomelosia crenata* (Cirillo) Greuter & Burdet, *Cynanchica aristata* (L.f.) P.Caputo & Del Guacchio subsp. *aristata*, and many others), seems not to have been much investigated yet in Mediterranean environments [104,105,106]. However, the phenomenon is related to the general characteristics of cliff habitats, whose flora is often determined primarily by elevation and distance from the sea, but where some species can exhibit unusual tolerance, sometimes coexisting with species with contrasting ecology. Such plasticity might be somehow reflected by the moderate capacity of *C. fragilis* to become spontaneous in central and northern Europe. In *C. fragilis* subsp. *cavolinii*, a preference for the colder and drier climate with respect to subsp. *fragilis* was observed, with a lower mean annual temperature range and precipitation regime in the driest and coldest months. Interestingly, the models also point out that the subspecies have the highest probability of occurrence, respectively, in the southern Abruzzo and Molise (in an area roughly corresponding to the National Park of Abruzzo, Lazio, and Molise) and in the peninsula of Sorrento in Campania, namely along the Amalfi Coast, and in western Calabria. These are the areas where the species was first observed and where, despite anthropization, it is still more common. From a conservation perspective, the species in the wild does not deserve particular concern, as both subspecies are assessed as “least concern” [107,108] due to local abundance, capability to colonize also urbanized environments, occurrence in low-disturbance areas, and their protection by local laws and national and regional protected areas (http://www.pcn.minambiente.it/viewer/index.php?services=progetto_natura; accessed on 25 August 2024). Nevertheless, according to our predicted distribution, particular attention should be paid to marginal populations of Lazio and Molise, which are more likely to disappear as a consequence of climatic changes in the mid- and long-term. The population of Mt. Orlando, for instance, has experienced a remarkable reduction in the number of individuals and distribution range from the 1980s to the present day (FM, pers. obs.). In addition, this locality is not among the areas with the highest habitat suitability in our predictions. Therefore, also considering its genetic characteristics, it deserves proper conservation measures.

## Figures and Tables

**Table 1:** List of the samples employed in the present study.

Taxon	Sampling Sites	Code
cavolinii	Abruzzo, Campanile di Pizzoferrato (Herb. Del Guacchio n. 701, NAP).	ABR1
cavolinii	Abruzzo, Scanno (this study) (41.94845° N, 13.82641° E)	ABR2
cavolinii	Abruzzo, Scanno (this study) (41.94845° N, 13.82641° E)	ABR3
cavolinii	Abruzzo, Scanno (this study) (41.94845° N, 13.82641° E)	ABR4
cavolinii	Lazio, Grotta dell’Inferniglio (RO, Herb. Anzalone n. 9761, sub *C. cavolinii*)	LAZ1
cavolinii	Lazio, Convento di S. Benedetto (RO, Herb. Anzalone n. 9752, sub *C. cavolinii*)	LAZ2
cavolinii	Lazio, Certosa di Trisulti (RO, Herb. Anzalone n. 9759, sub *C. fragilis s.l.*)	LAZ3
cavolinii	Molise, Pescopennataro (this study) (41.87908° N, 14.29308° E)	MOL1
cavolinii	Molise, Pescopennataro (this study) (41.87908° N, 14.29308° E)	MOL2
cavolinii	Molise, Pescopennataro (this study) (41.87908° N, 14.29308° E)	MOL3
fragilis	Basilicata, Maratea (Herb. Del Guacchio n. 3280, NAP)	BAS1
fragilis	Basilicata, Balvano (Herb. Del Guacchio n. 4455, NAP)	BAS2
fragilis	Basilicata, Timpa Falconara (CLU 1677)	BAS3
fragilis	Calabria, Laino Castello (CLU 1663)	CAL1
fragilis	Calabria, Mt. Cocuzzo (CLU 1673)	CAL2
fragilis	Calabria, Mounts of Verbicaro (CLU 1665)	CAL3
fragilis	Calabria, Passo Scalone (CLU 1676)	CAL4
fragilis	Calabria, Pazzano (CLU 1667)	CAL5
fragilis	Calabria, Timpa di Porace (CLU 1664)	CAL6
fragilis	Calabria, Timpa di Porace (CLU 1674)	CAL7
fragilis	Calabria, Orsomarso (CLU 1671)	CAL8
fragilis	Campania, Caggiano (this study) (40.568170° N, 15.486758° E)	CAM1
fragilis	Campania, Caggiano (this study) (40.568170° N, 15.486758° E)	CAM2
fragilis	Campania, Caggiano (this study) (40.568170° N, 15.486758° E)	CAM3
fragilis	Campania, Caggiano (this study) (40.568170° N, 15.486758° E)	CAM4
fragilis	Campania, Capri (this study) (40.54910° N, 14.25671° E)	CAM5
fragilis	Campania, Capri (this study) (40.54910° N, 14.25671° E)	CAM6
fragilis	Campania, Capri (this study) (40.54910° N, 14.25671° E)	CAM7
fragilis	Campania, Montevergine (this study) (40.935158° N, 14.728934° E)	CAM8
fragilis	Campania, Montevergine (this study) (40.935158° N, 14.728934° E)	CAM9
fragilis	Campania, Montevergine (this study) (40.935158° N, 14.728934° E)	CAM10
fragilis	Campania, Mt. Accellica (Herb. Del Guacchio n. 4128, NAP)	CAM11
fragilis	Campania, Mt. Finestra (Herb. Del Guacchio n. 7834, NAP)	CAM12
fragilis	Campania, Mts. Alburni (this study) (40.513539° N, 15.376591° E)	CAM13
fragilis	Campania, Mts. Alburni (this study) (40.513539° N, 15.376591° E)	CAM14
fragilis	Campania, Tramonti (this study) (40.719149° N, 14.618230° E)	CAM15
fragilis	Campania, Tramonti (this study) (40.719149° N, 14.618230° E)	CAM16
fragilis	Campania, Tramonti (this study) (40.719149° N, 14.618230° E)	CAM17
fragilis	Campania, Tramonti (this study) (40.719149° N, 14.618230° E)	CAM18
fragilis	Lazio, Mt. Orlando (RO, Herb. Anzalone n. 9756, sub *C. fragilis* subsp. *cavolinii*)	LAZ4
fragilis	Lazio, Mt. Orlando (this study) (41.209657° N, 13.573378° E)	LAZ5
fragilis	Lazio, Mt. Orlando (this study) (41.209657° N, 13.573378° E)	LAZ6
fragilis	Lazio, Mt. Orlando (this study) (41.209657° N, 13.573378° E)	LAZ7
*C. isophylla*	Liguria, Finalese (Savona), Val Ponci, 01/06/1989, R. Poggi s. n. (GDOR)	ISO1
*C. isophylla*	Liguria, Finalese (Savona), Val Ponci, 01/06/1989, R. Poggi s. n. (GDOR)	ISO2
*C. isophylla*	Liguria, Finalese (Savona), Val Ponci, 01/06/1989, R. Poggi s. n. (GDOR)	ISO3
*C. versicolor*	Botanical Garden of Naples (cultivated)	VER1

## Data Availability

The DNA sequences associated with this study are available in the International Nucleotide Sequence Databases (DDBJ, ENA, and GenBank) under the following accession numbers: LC843458-LC843504 (ITS), LC843505-LC843550 (*ndh*J-*trn*F), LC843551-LC843597 (*pet*B-*pet*D), LC843598-LC843643 (*pet*N-*psb*M), LC843644-LC843690 (*trn*H-*psb*A), and LC843691-LC843735 (*trn*F-*trn*L).

## Supplementary Materials

The following supporting information can be downloaded at: https://www.mdpi.com/article/10.3390/plants13223169/s1, Data S1: Nuclear alignment; Data S2: Chloroplast alignment; Data S3: Concatenated nuclear and chloroplast alignment; Data S4: Chloroplast haplotypes; Figure S1: Bayesian consensus tree of the nuclear (ITS) dataset; Figure S2: Bayesian consensus tree of the chloroplast dataset; Figure S3: Pearson’s correlation analysis on environmental variables; Figure S4: Training omission rate and predicted area (AUC) and receiver operating characteristic (ROC) curve; Figure S5: Response curves of the ten selected bioclimatic variables used in the MaxEnt model; Figure S6: Occurrence of *Campanula fragilis* on the geo-lithological layer; Table S1: List of primers and amplification conditions for nuclear and chloroplast markers; Table S2: Occurrence records of *Campanula fragilis* utilized for MaxEnt modeling and plotting of maps; Table S3: List of chloroplast haplotypes and distribution across samples.

## References

[1] Myers N., Mittermeier R.A., Mittermeier C.G., da Fonseca G.A.B., Kent J. (2000). Biodiversity hotspots for conservation priorities. Nature.

[2] Brugiapaglia E. (2017). Origine della flora e della vegetazione italiana. La flora in Italia.

[3] Thompson J.D. (2020). Plant Evolution in the Mediterranean: Insights for Conservation.

[4] Nieto Feliner G. (2014). Patterns and processes in plant phylogeography in the Mediterranean Basin. A review. Perspect. Plant Ecol. Evol. Syst..

[5] Di Biase L., Pace L., Mantoni C., Fattorini S. (2021). Variations in Plant Richness, Biogeographical Composition, and Life Forms along an Elevational Gradient in a Mediterranean Mountain. Plants.

[6] La Valva V. (1992). La *Aspetti corologici* della flora di interesse fitogeografico nell’Apennino Meridionale. Plant Biosyst..

[7] Brundu G., Peruzzi L., Domina G., Bartolucci F., Galasso G., Peccenini S., Raimondo F.M., Albano A., Alessandrini A., Banfi E. (2017). At the intersection of cultural and natural heritage: Distribution and conservation of the type localities of Italian endemic vascular plants. Biol. Conserv..

[8] Bartolucci F., Peruzzi L., Galasso G., Alessandrini A., Ardenghi N.M.G., Bacchetta G., Banfi E., Barberis G., Bernardo L., Bouvet D. (2024). A second update to the checklist of the vascular flora native to Italy. Plant Biosyst. Int. J. Deal. All Asp. Plant Biol..

[9] Mansion G., Parolly G., Crowl A.A., Mavrodiev E., Cellinese N., Oganesian M., Fraunhofer K., Kamari G., Phitos D., Haberle R. (2012). How to Handle Speciose Clades? Mass Taxon-Sampling as a Strategy towards Illuminating the Natural History of *Campanula* (Campanuloideae). PLoS ONE.

[10] POWO Plants of the World Online Facilitated by the Royal Botanic Gardens, Kew. https://powo.science.kew.org/.

[11] Greuter W., Burdeth M., Long G. (1984). Med-Checklist.

[12] Park J.M., Kovačić S., Liber Z., Eddie W.M.M., Schneeweiss G.M. (2006). Phylogeny and biogeography of isophyllous species of *Campanula* (Campanulaceae) in the Mediterranean area. Syst. Bot..

[13] Fedorov A.A., Tutin T.G., Heywood V.H., Burges N.A., Moore D.M., Valentine D.H., Walters S.M., Webb D.A. (1976). Campanulaceae Juss. Flora Europaea.

[14] Rafinesque C.S. (1813). Chloris Aetnensis.

[15] Presl K.B., Typographia J. (1831). Symbolae Botanicae.

[16] Nyman C.F. (1879). Conspectus Florae Europaeae.

[17] Avé-Lallemant J.L.E. (1829). De Plantis Quibusdam Italiae Borealis et Germaniae Australis Rarioribus.

[18] Gussone G. (1842). Florae Siculae Synopsis.

[19] Béguinot A., Fiori A., Paoletti G. (1903). Campanulaceae. Flora Analitica d’Italia.

[20] D’Errico P. (1947). Flora e boschi dell’Altipiano di Asiago. L’Italia For. Mont..

[21] Portal to the Flora of Italy. http://dryades.units.it/floritaly.

[22] Scortegagna S. (2008). Flora del settore veneto dell’Altopiano di Asiago (Prealpi orientali, provincia di Vicenza). Nat. Vicentina.

[23] Rodegher E., Rodegher A. (1929). Flora della Prov. di Bergamo. V puntata. Atti Ateneo Sci. Lett. Arti Bergamo (1927–1929). Bergomum Bollettino Della Civica Biblioteca.

[24] Martini F. (2012). Flora Vascolare Della Lombardia Centro-Orientale.

[25] Cirillo D.M.L. (1788). Plantarum Rariorum Regni Neapolitani.

[26] Moggridge J.T. (1871). Contributions to the Flora of Mentone and to a Winter Flora of the Riviera, Including the Coast from Marseilles to Genoa.

[27] Lewis P., Lynch M. (1998). Campanulas: A gardener’s Guide.

[28] Eddie W.M.M., Cann D.C.G., Cullen J., Knees S.G., Cubey H.S. (2011). *Campanula* Linnaeus. The European Garden Flora. V. Boraginaceae to Compositae.

[29] Jeffery M. (2024). The Kew Gardener’s Guide to Growing Alpines.

[30] Bernardo L., Peruzzi L. (2011). Notulae alla checklist della Flora vascolare Italiana 12 (1823–1883). Inf. Bot. Ital..

[31] Preston C.D., Pearman D.A., Dines T.D. (2002). New Atlas of the British Isles Flora.

[32] Sell P., Murrell G. (2006). Flora of Great Britain and Ireland: Volume 4, Campanulaceae—Asteraceae.

[33] Buttler K.P., Thieme M. Mitarbeiter Florenliste von Deutschland—Gefäßpflanzen. Version 10. http://www.kp-buttler.de/florenliste/index.htm.

[34] Wasowicz P., Przedpelska-Wasowicz E.M., Kristinsson H. (2013). Alien vascular plants in Iceland: Diversity, spatial patterns, temporal trends, and the impact of climate change. Flora Morphol. Distrib. Funct. Ecol. Plants.

[35] Frizzi G., Tammaro F. (1991). Electrophoretic study and genetic affinity in the *Campanula elatines* and *C. fragilis* (Campanulaceae) rock-plants group from Italy and W. Jugoslavia. Plant Syst. Evol..

[36] Damboldt J. (1965). Zytotaxonomische Revision der isophyllen Campanulae in Europa. Bot. Jahrb. Syst. Pflanzengesch. Pflanzengeogr..

[37] Pignatti S. (1982). Flora d’Italia.

[38] Bartolucci F., Peruzzi L., Galasso G., Albano A., Alessandrini A., Ardenghi N.M.G., Astuti G., Bacchetta G., Ballelli S., Banfi E. (2018). An updated checklist of the vascular flora native to Italy. Plant Biosyst. Int. J. Deal. All Asp. Plant Biol..

[39] Arata M. (1938). Contributo Allo Studio Della Flora del Cilento (Salernitano). G. Bot. Ital..

[40] Moraldo B., Minutillo F., Rossi W. (1990). Flora del Lazio Meridionale. Quad. Dell’accademia Naz. Dei Lincei.

[41] Lucchese F. (1993). *Campanula reatina*, a new species restricted to some cliffs in the Sabina area (Lazio, central Italy). Flora Mediterr..

[42] Anzalone B., Iberite M., Lattanzi E. (2010). La flora vascolare del Lazio. Inf. Bot. Ital..

[43] Lucchese F. (2018). Atlante della Flora Vascolare del Lazio, cartografia, ecologia e biogeografia. La Flora di Maggiore Interesse Conservazionistico.

[44] Castroviejo S., Aldasoro J.J., Alarcón M. Campanulaceae. https://europlusmed.org/cdm_dataportal/taxon/9a4a0532-431a-47eb-bd4f-66c9d523748e/synonymy?highlight=aa6c0b5f-edd7-4a5d-a66e-d80c8c03a80c&acceptedFor=aa6c0b5f-edd7-4a5d-a66e-d80c8c03a80c#aa6c0b5f-edd7-4a5d-a66e-d80c8c03a80c.

[45] WFO *Campanula fragilis* Cirillo. http://www.worldfloraonline.org/taxon/wfo-0001290031.

[46] Tenore M. (1824–1829). Flora Napolitana.

[47] Bertoloni A. (1835). Flora Italica.

[48] Fiori A. (1927). Nuova Flora Analitica d’Italia.

[49] Lovašen-Eberhardt Z., Trinajstic I. (1978). O geografskoj distribuciji morfoloških karakteristika vrsta serije Garganicae roda *Campanula* L. u flori Jugoslavije. Biosistematika.

[50] Pignatti S. (2018). Flora d’Italia.

[51] Damboldt J. (1968). Kurzer Nachtrag zur “Zytotaxonomischen Revision der isophyllen Campanulae in Europa”. Bot. Jahrbücher Syst. Pflanzengesch. Pflanzengeogr..

[52] Kovačić S. (2004). The genus *Campanula* L. (Campanulaceae) in Croatia, circum-Adriatic and west Balkan region. Acta Bot. Croat..

[53] Liber Z., KovaČić S., Nikolić T., Likić S., Rusak G. (2008). Relations between western Balkan endemic *Campanula* L. (Campanulaceae) lineages: Evidence from chloroplast DNA. Plant Biosyst. Int. J. Deal. All Asp. Plant Biol..

[54] Frajman B., Schneeweiss G.M. (2009). A Campanulaceous Fate: The Albanian Stenoendemic *Asyneuma comosiforme* in fact Belongs to Isophyllous *Campanula*. Syst. Bot..

[55] Bogdanović S., Brullo S., Rešetnik I., Satovic Z., Liber Z. (2014). *Campanula teutana*, a new isophyllous *Campanula* (Campanulaceae) from the Adriatic region. Phytotaxa.

[56] Bogdanović S., Brullo S., Rešetnik I., Lakušić D., Satovic Z., Liber Z. (2014). *Campanula skanderbegii*: Molecular and Morphological Evidence of a New *Campanula* Species (Campanulaceae) Endemic to Albania. Syst. Bot..

[57] Bogdanović S., Rešetnik I., Brullo S., Shuka L. (2015). *Campanula aureliana* (Campanulaceae), a new species from Albania. Plant Syst. Evol..

[58] Bogdanović S., Rešetnik I., Jeričević M., Jeričević N., Brullo S. (2019). Molecular and morphological survey on *Campanula cremnophila* (Campanulaceae), a new isophyllous species from Croatia. Plant Syst. Evol..

[59] Rešetnik I., Temunović M., Liber Z., Satovic Z., Bogdanović S. (2020). Phylogeography of *Campanula fenestrellata* s.l. (Campanulaceae) in the northern Adriatic. Plant Syst. Evol..

[60] Xu C., Hong D.-Y. (2021). Phylogenetic analyses confirm polyphyly of the genus *Campanula* (Campanulaceae s. str.), leading to a proposal for generic reappraisal. J. Syst. Evol..

[61] Dong W., Liu J., Yu J., Wang L., Zhou S. (2012). Highly Variable Chloroplast Markers for Evaluating Plant Phylogeny at Low Taxonomic Levels and for DNA Barcoding. PLoS ONE.

[62] Starr J.R., Harris S.A., Simpson D.A. (2003). Potential of the 5′ and 3′ Ends of the Intergenic Spacer (IGS) of rDNA in the Cyperaceae: New Sequences for Lower-Level Phylogenies in Sedges with an Example from *Uncinia* Pers. Int. J. Plant Sci..

[63] Aceto S., Caputo P., Cozzolino S., Gaudio L., Moretti A. (1999). Phylogeny and Evolution of *Orchis* and Allied Genera Based on ITS DNA Variation: Morphological Gaps and Molecular Continuity. Mol. Phylogenet. Evol..

[64] Shaw J., Lickey E.B., Schilling E.E., Small R.L. (2007). Comparison of whole chloroplast genome sequences to choose noncoding regions for phylogenetic studies in angiosperms: The tortoise and the hare III. Am. J. Bot..

[65] Prince L.M. (2015). Plastid primers for angiosperm phylogenetics and phylogeography. Appl. Plant Sci..

[66] Lee C., Wen J. (2004). Phylogeny of *Panax* using chloroplast *trn*C–*trn*D intergenic region and the utility of *trn*C–*trn*D in interspecific studies of plants. Mol. Phylogenet. Evol..

[67] Tate J.A., Simpson B.B. (2003). Paraphyly of *Tarasa* (Malvaceae) and Diverse Origins of the Polyploid Species. Syst. Bot..

[68] Sang T., Crawford D.J., Stuessy T.F. (1997). Chloroplast DNA phylogeny, reticulate evolution, and biogeography of *Paeonia* (Paeoniaceae). Am. J. Bot..

[69] Taberlet P., Gielly L., Pautou G., Bouvet J. (1991). Universal primers for amplification of three non-coding regions of chloroplast DNA. Plant Mol. Biol..

[70] Thompson J.D., Higgins D.G., Gibson T.J. (1994). CLUSTAL W: Improving the sensitivity of progressive multiple sequence alignment through sequence weighting, position-specific gap penalties and weight matrix choice. Nucleic Acids Res..

[71] Hall T.A. (1999). BioEdit: A user-friendly biological sequence alignment editor and analysis program for Windows 95/98/NT. Nucleic Acids Symp. Ser..

[72] Schloss P.D., Westcott S.L., Ryabin T., Hall J.R., Hartmann M., Hollister E.B., Lesniewski R.A., Oakley B.B., Parks D.H., Robinson C.J. (2009). Introducing mothur: Open-source, platform-independent, community-supported software for describing and comparing microbial communities. Appl. Environ. Microbiol..

[73] R Core Team (2020). R: A Language and Environment for Statistical Computing.

[74] Wickham H. (2016). ggplot2: Elegant Graphics for Data Analysis.

[75] Cheng J., Schloerke B., Karambelkar B., Xie Y. (2023). Leaflet: Create Interactive Web Maps with the JavaScript ‘Leaflet’ Library; R Package version 2.2.0. https://CRAN.R-project.org/package=leaflet.

[76] Darriba D., Taboada G.L., Doallo R., Posada D. (2012). jModelTest 2: More models, new heuristics and parallel computing. Nat. Methods.

[77] Akaike H. (1974). A new look at the statistical model identification. IEEE Trans. Automat. Contr..

[78] Goloboff P. (1999). NONA. Computer Software and Documentation, Version 2.0.

[79] Nixon K.C. (2002). WinClada.

[80] Ronquist F., Teslenko M., van der Mark P., Ayres D.L., Darling A., Höhna S., Larget B., Liu L., Suchard M.A., Huelsenbeck J.P. (2012). MrBayes 3.2: Efficient Bayesian phylogenetic inference and model choice across a large model space. Syst. Biol..

[81] Stöver B.C., Müller K.F. (2010). TreeGraph 2: Combining and visualizing evidence from different phylogenetic analyses. BMC Bioinform..

[82] Phillips S.J., Anderson R.P., Schapire R.E. (2006). Maximum entropy modeling of species geographic distributions. Ecol. Modell..

[83] Hijmans R. (2023). raster: Geographic Data Analysis and Modeling.

[84] Hijmans R.J., Phillips S., Leathwick J., Elith J. (2023). dismo: Species Distribution Modeling.

[85] Barbet-Massin M., Jiguet F., Albert C.H., Thuiller W. (2012). Selecting pseudo-absences for species distribution models: How, where and how many?. Methods Ecol. Evol..

[86] Fielding A.H., Bell J.F. (1997). A review of methods for the assessment of prediction errors in conservation presence/absence models. Environ. Conserv..

[87] Warren D.L., Seifert S.N. (2011). Ecological niche modeling in Maxent: The importance of model complexity and the performance of model selection criteria. Ecol. Appl..

[88] Fois M., Cuena-Lombraña A., Fenu G., Bacchetta G. (2018). Using species distribution models at local scale to guide the search of poorly known species: Review, methodological issues and future directions. Ecol. Modell..

[89] Sommacal N.S., Massidda D. (2015). mapIT: Maps of Italy.

[90] Bivand R., Keitt T., Rowlingson B. (2023). rgdal: Bindings for the ‘Geospatial’ Data Abstraction Library.

[91] Abdelaal M., Fois M., Fenu G., Bacchetta G. (2019). Using MaxEnt modeling to predict the potential distribution of the endemic plant *Rosa arabica* Crép. in Egypt. Ecol. Inform..

[92] Gargiulo R., Guacchio E.D., Caputo P. (2015). Phylogenetic reconstruction of *Asperula* sect. *Cynanchicae* (Rubiaceae) reveals a mosaic of evolutionary histories. Taxon.

[93] Nge F.J., Biffin E., Thiele K.R., Waycott M. (2021). Reticulate Evolution, Ancient Chloroplast Haplotypes, and Rapid Radiation of the Australian Plant Genus *Adenanthos* (Proteaceae). Front. Ecol. Evol..

[94] Rose J.P., Toledo C.A.P., Lemmon E.M., Lemmon A.R., Sytsma K.J. (2021). Out of Sight, Out of Mind: Widespread Nuclear and Plastid-Nuclear Discordance in the Flowering Plant Genus *Polemonium* (Polemoniaceae) Suggests Widespread Historical Gene Flow Despite Limited Nuclear Signal. Syst. Biol..

[95] De Luca D., Del Guacchio E., Conti F., Iamonico D., Caputo P. (2022). Relationships within *Mcneillia* Indicate a Complex Evolutionary History and Reveal a New Species of *Minuartiella* (Caryophyllaceae, Alsinoideae). Plants.

[96] Béguinot A., Fiori A., Béguinot A. (1911). 1565. *Campanula Cavolinii* Ten. var. *glabra* Ten. Flora Italica Exsiccate.

[97] Ray J. (1694). Stirpium europaearum Extra Britannias nascentium Sylloge.

[98] Tenore M. (1831). Sylloge Plantarum Vascularium Floræ Neapolitanae Hucusque Detectarum.

[99] Conti F., Abbate G., Alessandrini A., Blasi C. (2005). An Annotated Checklist of the Italian Vascular flora.

[100] Schouw J.F. (1824). Prospetto di una descrizione geografica delle piante d’Italia e di Sicilia, con un saggio di monigrafia delle specie del genere *Campanula* indigene d’Italia. G. Fis. Pavia.

[101] Béguinot A., Fiori A., Béguinot A. (1910). 1144. *Campanula fragilis* Cyr. var. *glabra* Ten. Flora Italica Exsiccate.

[102] Del Guacchio E., Caputo P. (2008). *Crocus imperati* and *Crocus suaveolens* (Iridaceae), two controversial endemic crocuses from Central and Southern Italy—Morphometrics, lectotypification and chorology. Bot. J. Linn. Soc..

[103] Croce A., La Valva V., Motti R., Nazzaro R., Strumia S. (2008). La flora vascolare del Vulcano di Roccamonfina (Campania, Italia). Webbia.

[104] Aronne G., Buonanno M., De Micco V. (2015). Reproducing under a warming climate: Long winter flowering and extended flower longevity in the only Mediterranean and maritime *Primula*. Plant Biol..

[105] Capó M., Cortés-Fernández I., Borràs J. (2023). Spatial distribution of insular cliff vegetation and future scenarios in a climate change perspective. bioRxiv.

[106] Borràs J., Cortés-Fernández I., Capó M. (2024). Spatial distribution of cliff plant species in the Balearic Islands under current and projected climatic scenarios. Basic Appl. Ecol..

[107] Rossi G., Orsenigo S., Gargano D., Montagnani C., Peruzzi L., Fenu G., Abeli T., Alessandrini A., Astuti G., Bacchetta G. (2020). Lista Rossa della Flora Italiana. 2 Endemiti e altre Specie Minacciate.

[108] Conti F., Tinti D., Bartolucci F. (2024). Plants of Conservation Interest in a Protected Area: A Case Study of the Gran Sasso and Monti Della Laga National Park (Central Italy). Plants.

