# Supplementary material for: Genetics and Distribution of the Italian Endemic Campanula fragilis Cirillo (Campanulaceae)
Source: Plants (Basel). 2024 Nov 11;13(22):3169. doi: 10.3390/plants13223169 (PMC11598242; doi:10.3390/plants13223169)
Supplement: Supplementary file 1 [file plants-13-03169-s001.zip › Figure S3.pdf]

Figure S3. Pearson's correlation analysis on environmental variables. (a) Heatmap with correlation's values; (b) cluster analysis of correlated variables at Pearson's correlation > 0.8.

A

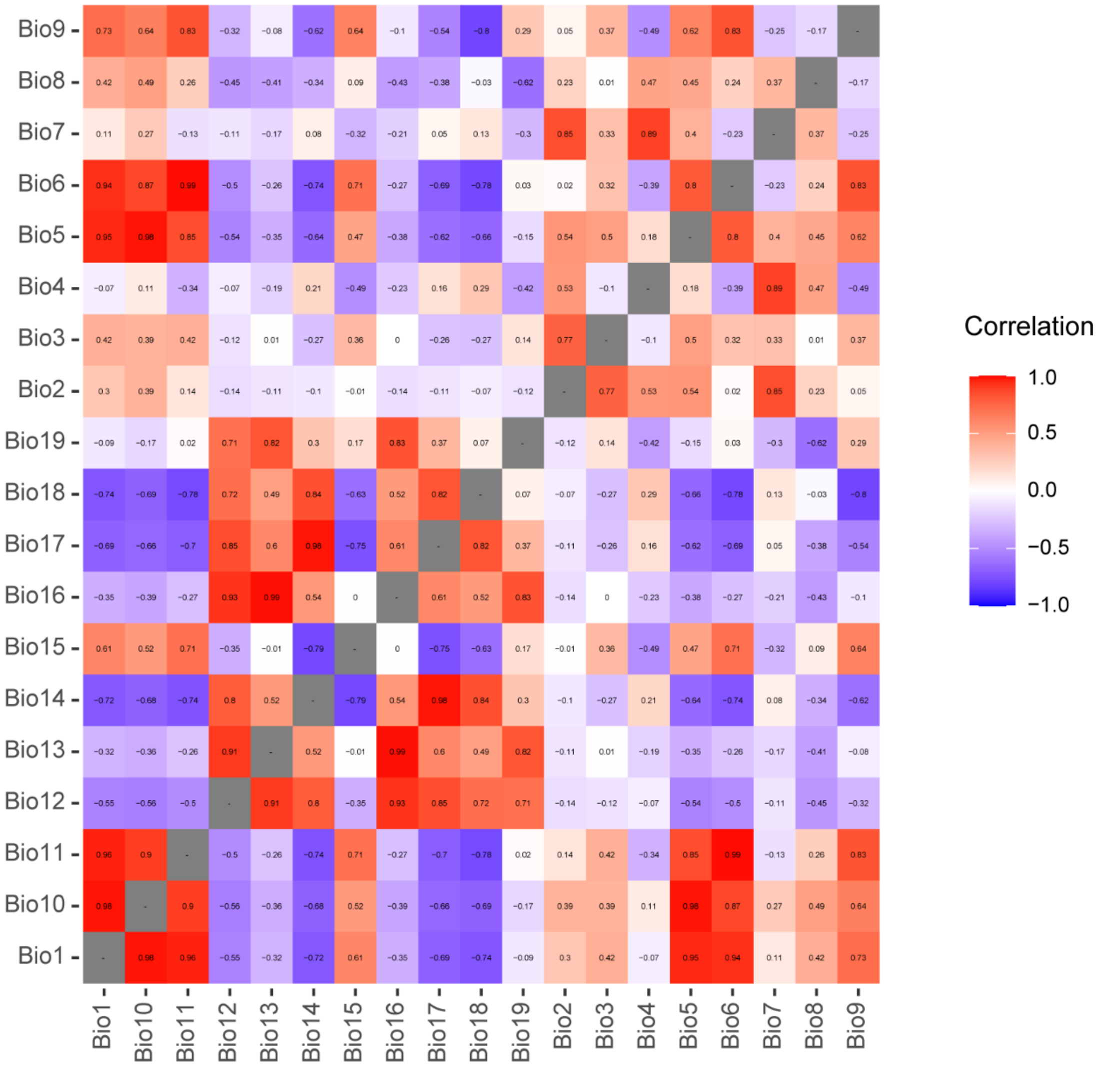

B

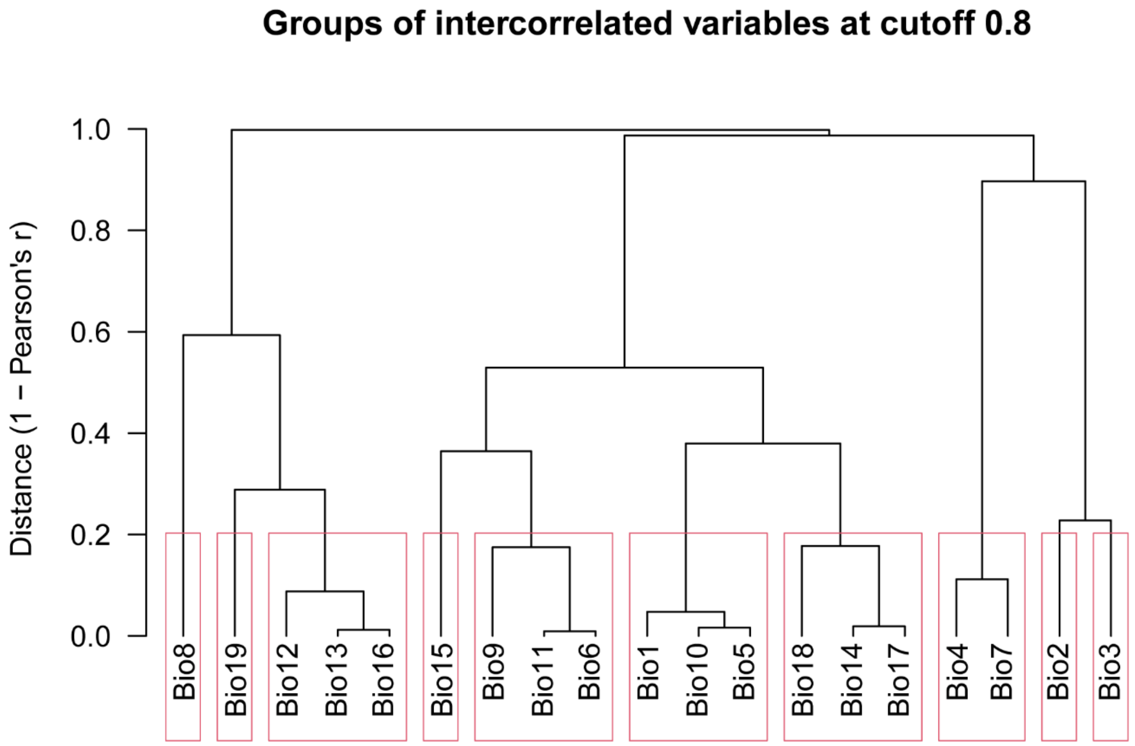
Groups of intercorrelated variables at cutoff 0.8
Distance (1 − Pearson's r)
1.0
0.8
0.6
0.4
0.2
0.0
Bio8
Bio19
Bio12
Bio13
Bio16
Bio15
Bio9
Bio11
Bio6
Bio1
Bio10
Bio5
Bio18
Bio14
Bio17
Bio4
Bio7
Bio2
Bio3
