# Supplementary material for: Genetics and Distribution of the Italian Endemic Campanula fragilis Cirillo (Campanulaceae)
Source: Plants (Basel). 2024 Nov 11;13(22):3169. doi: 10.3390/plants13223169 (PMC11598242; doi:10.3390/plants13223169)

Figure S4. Training omission rate and predicted area (AUC), and receiver operating characteristic (ROC) curve. (a) AUC for *Campanula fragilis* subsp. *cavolinii*; (b) ROC for the same subspecies; (c) AUC for *C. fragilis* subsp. *fragilis*; (d) ROC for the same subspecies. Data are averaged over the replicate runs (10).

A

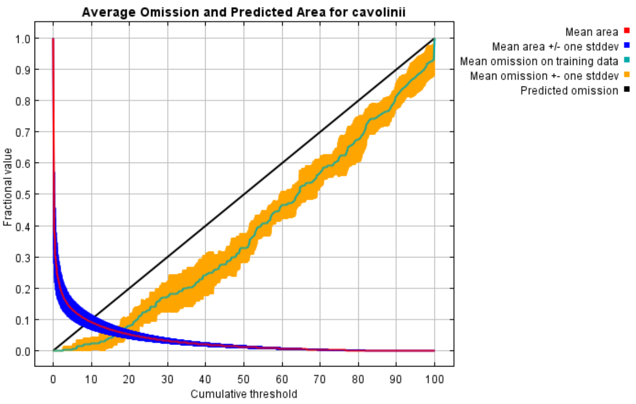

B

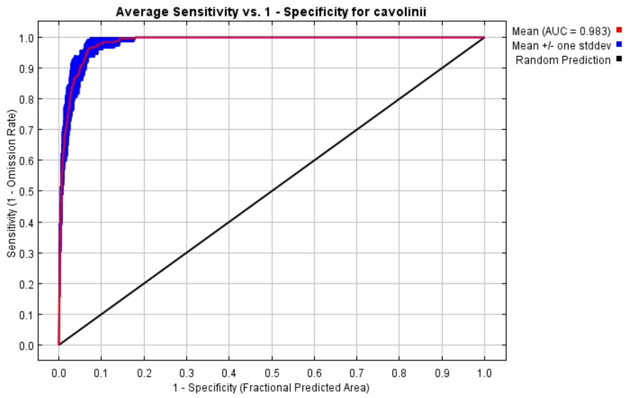

C

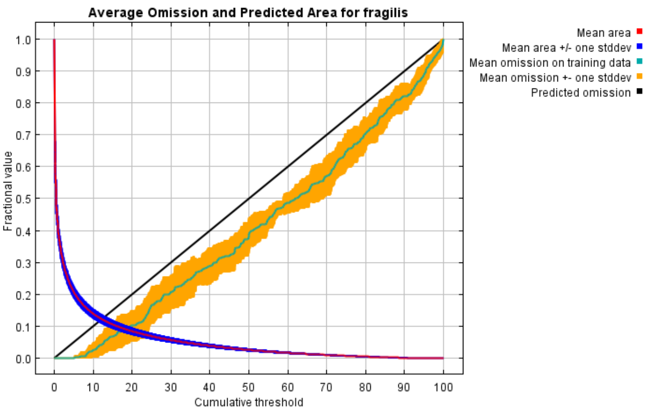

D

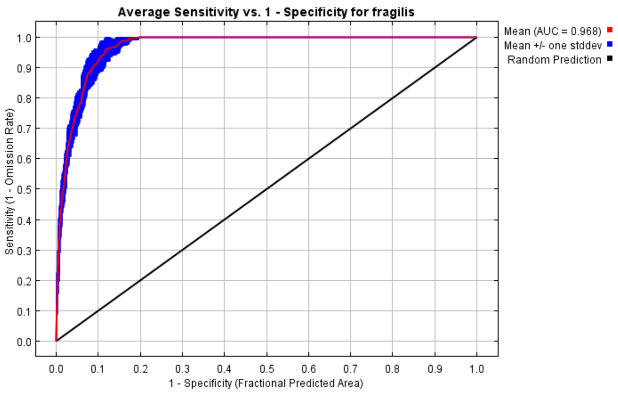

Supplement: Supplementary file 1 [file plants-13-03169-s001.zip › Figure S4.pdf]
