# Supplementary material for: Genetics and Distribution of the Italian Endemic Campanula fragilis Cirillo (Campanulaceae)
Source: Plants (Basel). 2024 Nov 11;13(22):3169. doi: 10.3390/plants13223169 (PMC11598242; doi:10.3390/plants13223169)
Supplement: Supplementary file 1 [file plants-13-03169-s001.zip › Figure S5.pdf]

Figure S5. Response curves of the ten selected bioclimatic variables used in the MaxEnt model. (a) *Campanula fragilis* subsp. *cavolinii*; (b) *C. fragilis* subsp. *fragilis*.

A

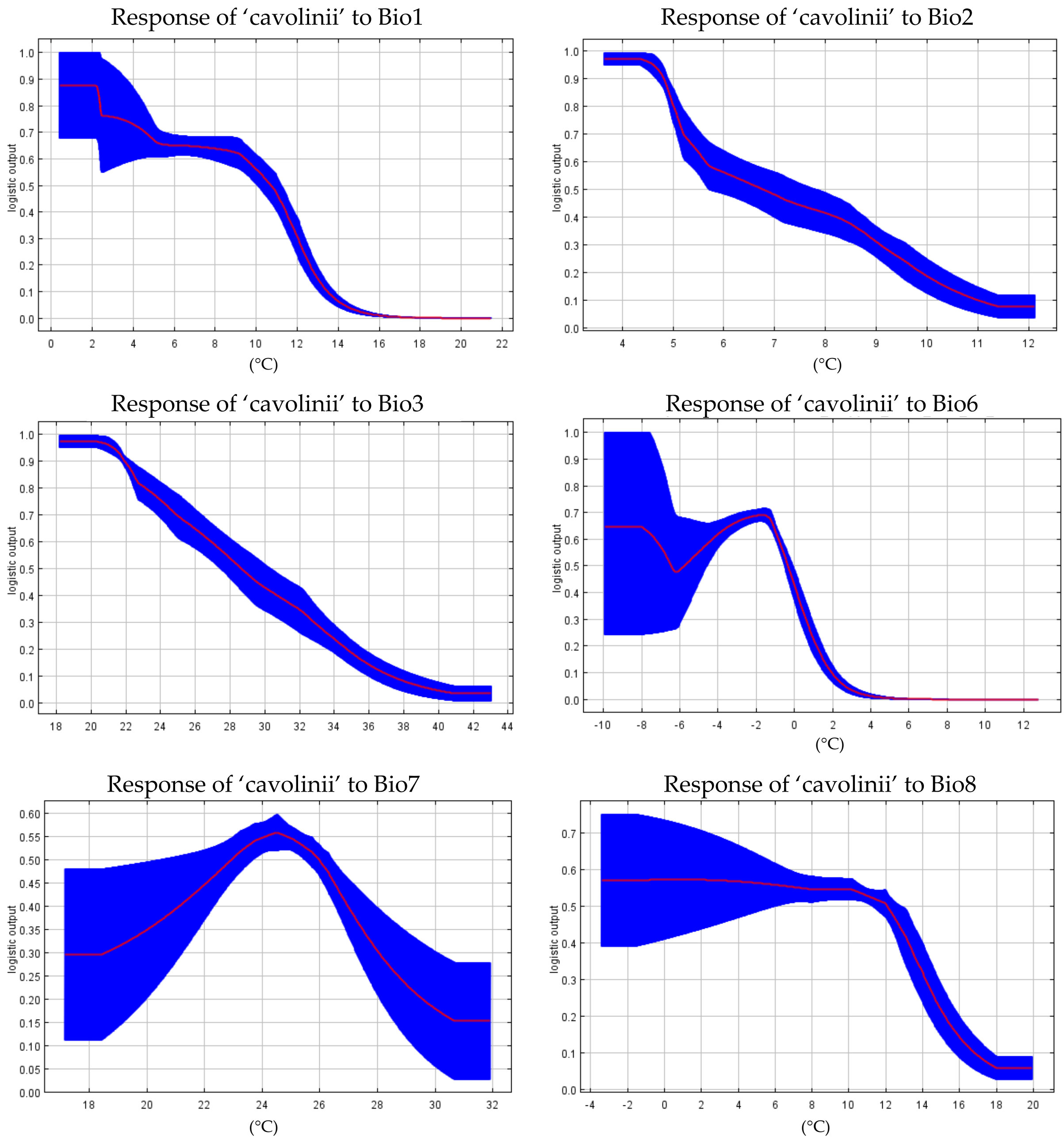

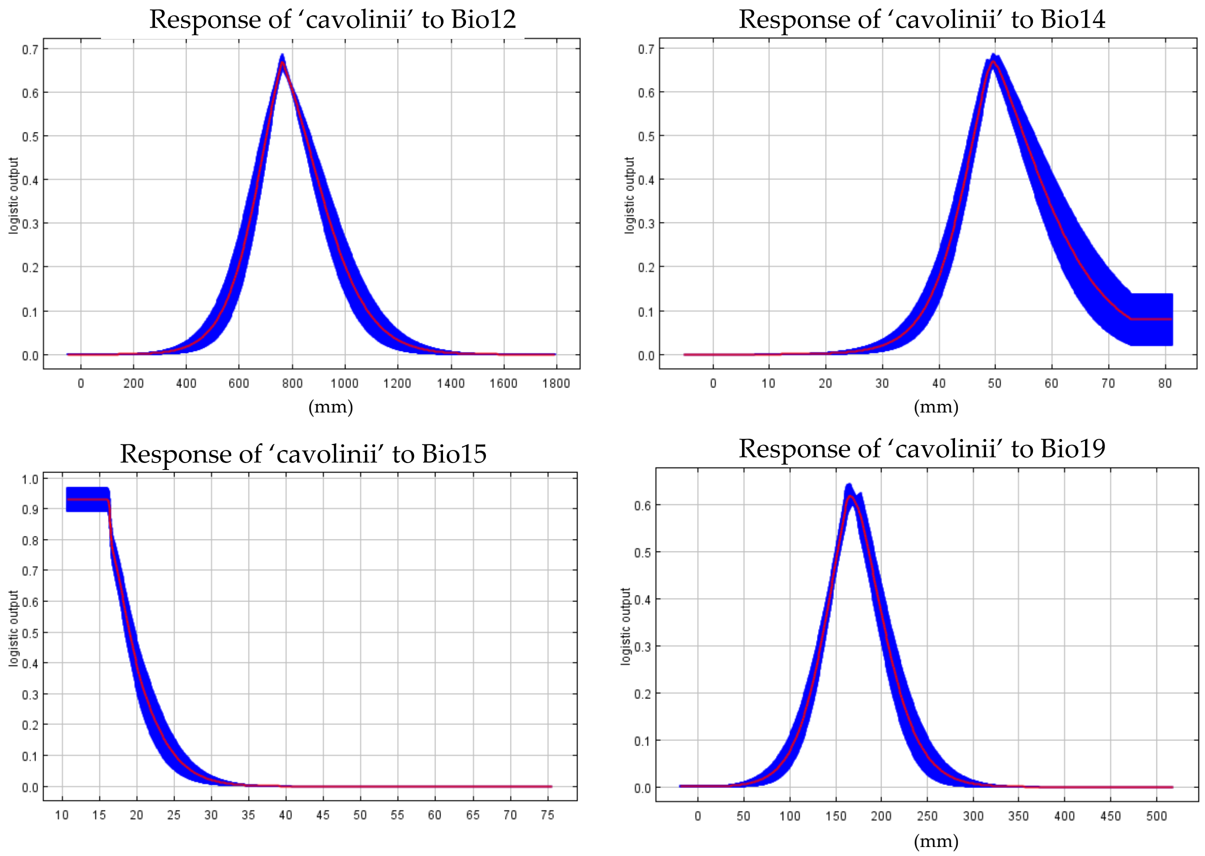
Response of 'cavolinii' to Bio12
Response of 'cavolinii' to Bio14
Response of 'cavolinii' to Bio15
Response of 'cavolinii' to Bio19
logistic output
(mm)

B

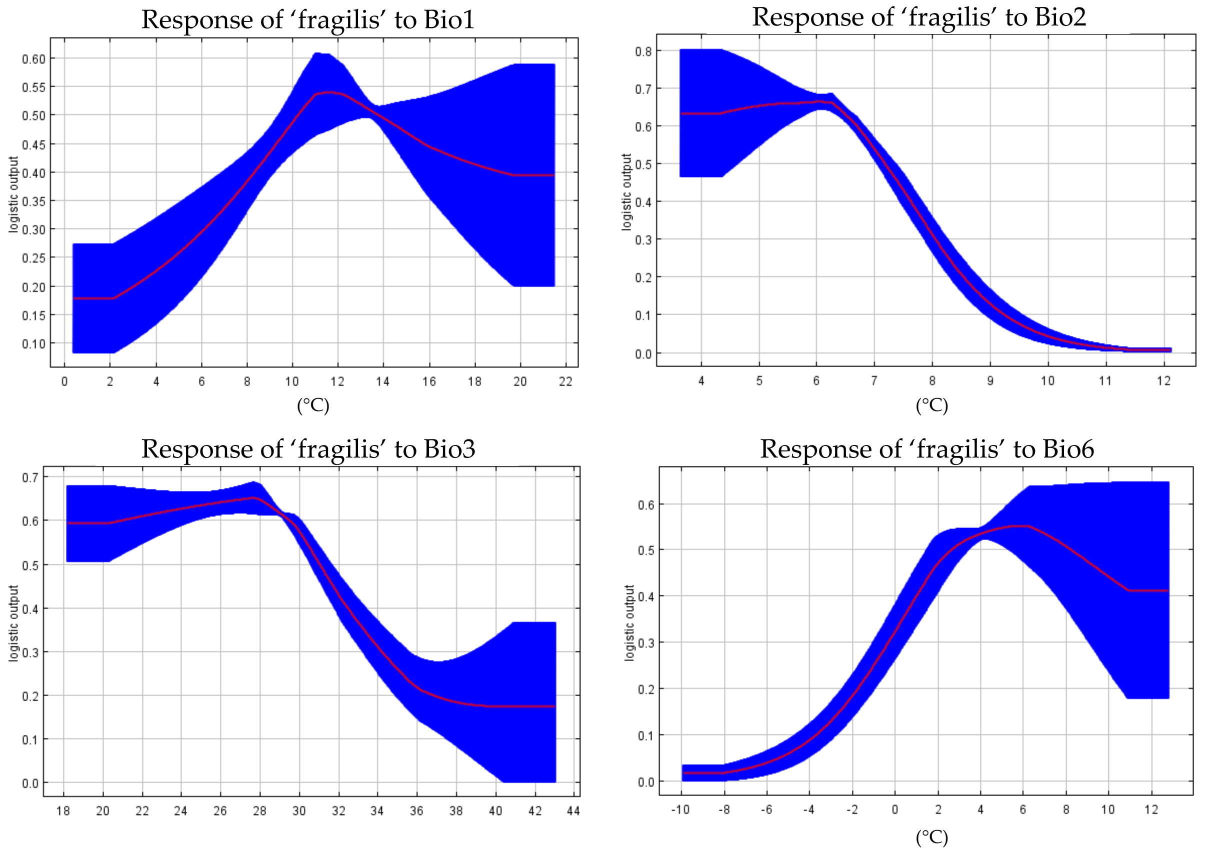
Response of 'fragilis' to Bio1
Response of 'fragilis' to Bio2
Response of 'fragilis' to Bio3
Response of 'fragilis' to Bio6
logistic output
(°C)

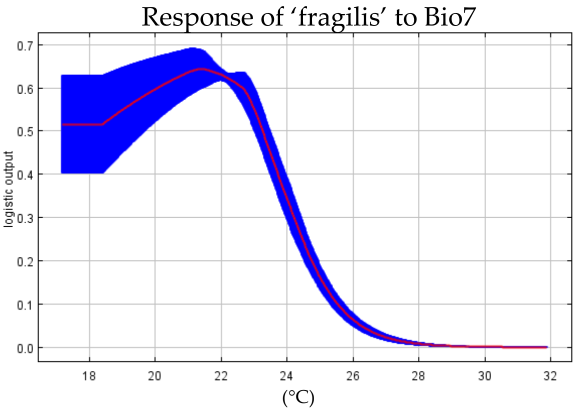
Response of 'fragilis' to Bio7
logistic output
(°C)

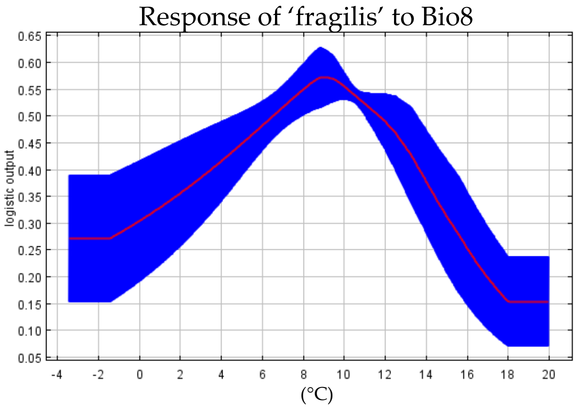
Response of 'fragilis' to Bio8
logistic output
(°C)

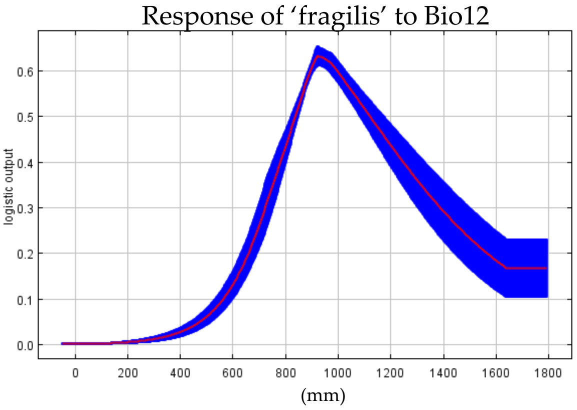
Response of 'fragilis' to Bio12
logistic output
(mm)

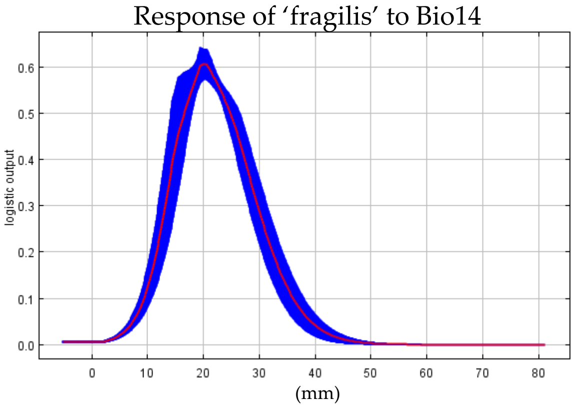
Response of 'fragilis' to Bio14
logistic output
(mm)

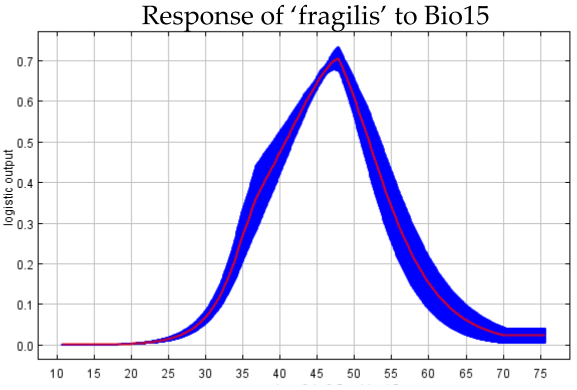
Response of 'fragilis' to Bio15
logistic output

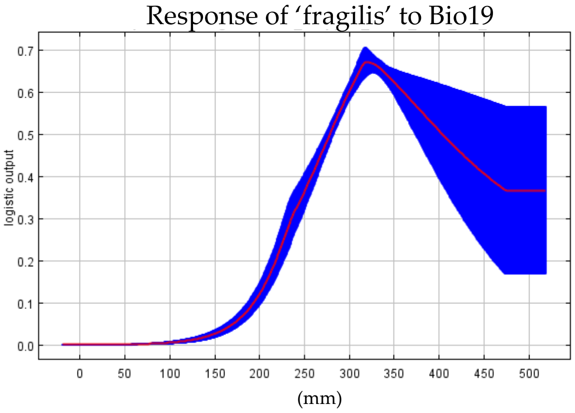
Response of 'fragilis' to Bio19
logistic output
(mm)
