# Supplementary material for: Genetics and Distribution of the Italian Endemic Campanula fragilis Cirillo (Campanulaceae)
Source: Plants (Basel). 2024 Nov 11;13(22):3169. doi: 10.3390/plants13223169 (PMC11598242; doi:10.3390/plants13223169)

Figure S6. Occurrence of *Campanula fragilis* on geo-lithological layer. (a) *C. fragilis* subsp. *cavolinii*; (b) *C. fragilis* subsp. *fragilis*.

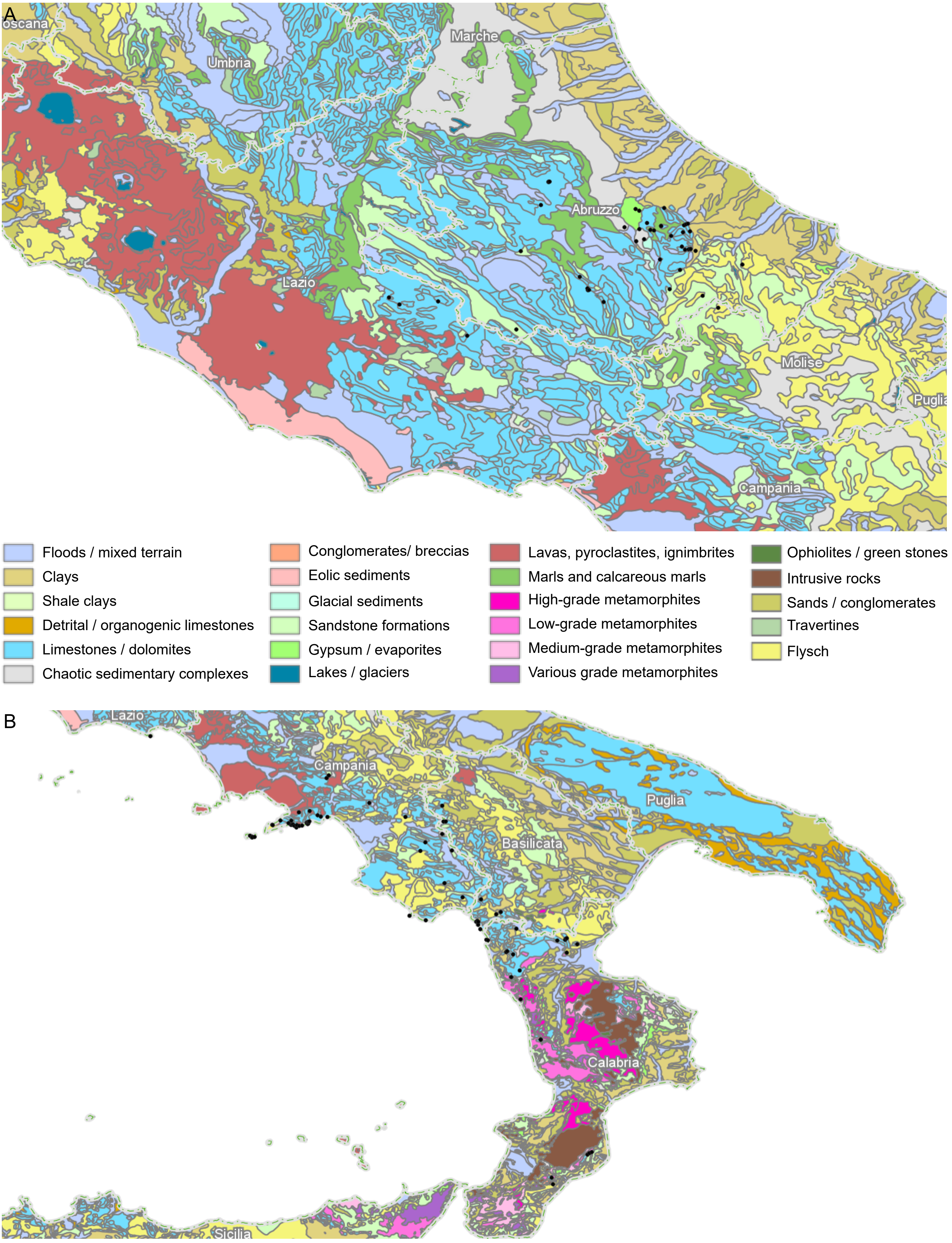

Supplement: Supplementary file 1 [file plants-13-03169-s001.zip › Figure S6.pdf]
