# Supplementary material for: Genetics and Distribution of the Italian Endemic Campanula fragilis Cirillo (Campanulaceae)
Source: Plants (Basel). 2024 Nov 11;13(22):3169. doi: 10.3390/plants13223169 (PMC11598242; doi:10.3390/plants13223169)
Supplement: Supplementary file 1 [file plants-13-03169-s001.zip › Table S1.pdf]

Table S1. List of primers and amplification conditions for nuclear and chloroplast markers.

Genome	Marker	Oligo name	Primer sequence (5’-3’)	Reference	PCR program
nuclear	ITS	JK14	GGAGAAGTCGTAACAAGGTTTCCG	Aceto et al. (1999)	A
		JK12	CCAAACAACCCGACTCGTAGACAGC	Aceto et al. (1999)	
chloroplast	*ndh*J-*trn*F	ndhJ	ATGCCYGAAAGTTGGATAGG	Shaw et al. (2007)	B
		trnF-IGSF	ATCCTCGTGTCACCAGTTCAAA	Prince (2015)	
	*pet*B-*pet*D	petBE2-IGSF	ATGCACTTTCCAATGATACG	Prince (2015)	A
		petD-E2R	CCCGAGGGAACCGGACAT	Prince (2015)	
	*pet*N-*psb*M	petN1	GGATATAGTAAGTCTTGCTTGGG	Lee and Wen (2004)	A
		psbM2R	TTCTTGCATTTATTGCTACTGC	Lee and Wen (2004)	
	*trn*H-*psb*A	trnHf	CGCGCATGGTGGATTCACAATCC	Tate et al. 2003	B
		psbA3f	GTTATGCATGAACGTAATGCTC	Sang et al. (1997)	
	*trn*F-*trn*L	trnF1(e)	GGTTCAAGTCCCTCTATCCC	Taberlet et al. (1991)	B
		trnF2(f)	ATTTGAACTGGTGACACGAG	Taberlet et al. (1991)	

Program	Initial denaturation	Denaturation	Annealing	Extension	Final extension	Notes
A	94°C, 3 min	94°C, 30 s	See note, 30 s	72°C, 1 min 30 s	7 min	T_a ITS = 62°C; T_a *pet*B-*pet*D = 58°C; T_a *pet*N-*psb*M = 53°C
		x 35 cycles				
B	94°C, 3 min	94°C, 30 s	See note, 30 s	72°C, 1 min	1 min	T_a *ndh*J-*trn*F = 53°C; T_a *trn*H-*psb*A, *trn*F-*trn*L= 55°C;
		x 35 cycles				
