# Supplementary material for: Genetics and Distribution of the Italian Endemic Campanula fragilis Cirillo (Campanulaceae)
Source: Plants (Basel). 2024 Nov 11;13(22):3169. doi: 10.3390/plants13223169 (PMC11598242; doi:10.3390/plants13223169)
Supplement: Supplementary file 1 [file plants-13-03169-s001.zip › Table S3.pdf]

Table S3. List of chloroplast haplotypes and distribution across samples.

Haplotype	Reference sample	Corresponding samples
H1	LAZ2	LAZ2
H2	LAZ1	LAZ1
H3	ABR2	ABR2, ABR3, ABR4
H4	ABR1	ABR1
H5	MOL1	MOL1, MOL2, MOL3
H6	LAZ3	LAZ3, LAZ4, LAZ5, LAZ6, LAZ7, CAM8, CAM9, CAM10, CAM11, CAM12, CAM15, CAM16, CAM17, CAM18
H7	CAM5	CAM5, CAM6, CAM7
H8	CAM13	CAM13, CAM14
H9	BAS2	BAS2
H10	CAM1	CAM1, CAM2, CAM3, CAM4
H11	BAS1	BAS1, CAL1, CAL3, CAL4
H12	BAS3	BAS3, CAL2
H13	CAL6	CAL6
H14	CAL7	CAL7
H15	CAL8	CAL8
H16	CAL5	CAL5
