# Supplementary figures and images for: Genetics and Distribution of the Italian Endemic Campanula fragilis Cirillo (Campanulaceae)
Source: Plants (Basel). 2024 Nov 11;13(22):3169. doi: 10.3390/plants13223169 (PMC11598242; doi:10.3390/plants13223169)

**Figure S1.** Bayesian consensus tree of the nuclear (ITS) dataset.

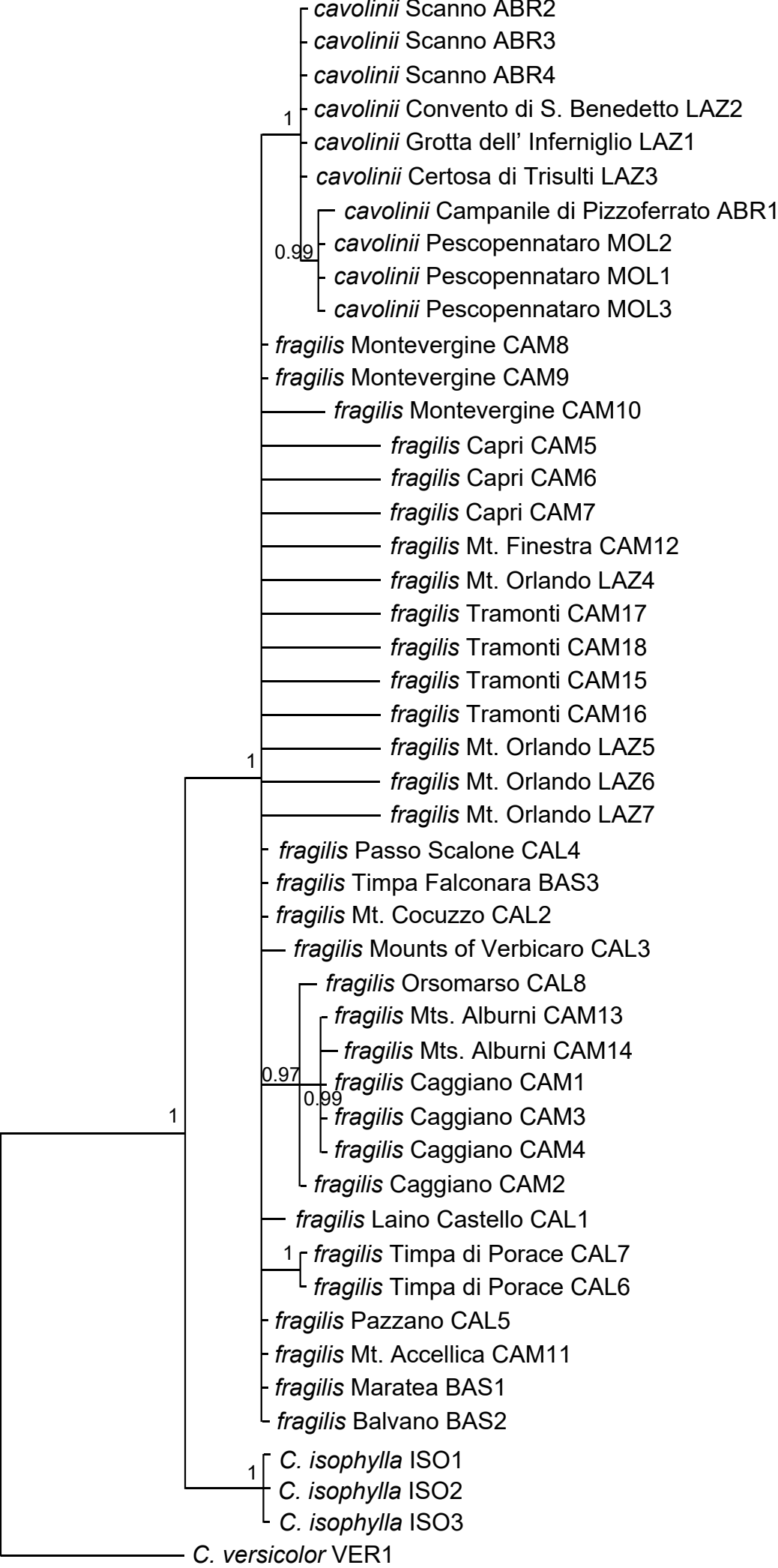

0.002

Supplement: Supplementary file 1 [file plants-13-03169-s001.zip › Figure S1.pdf]

**Figure S2.** Bayesian consensus tree of the chloroplast dataset.

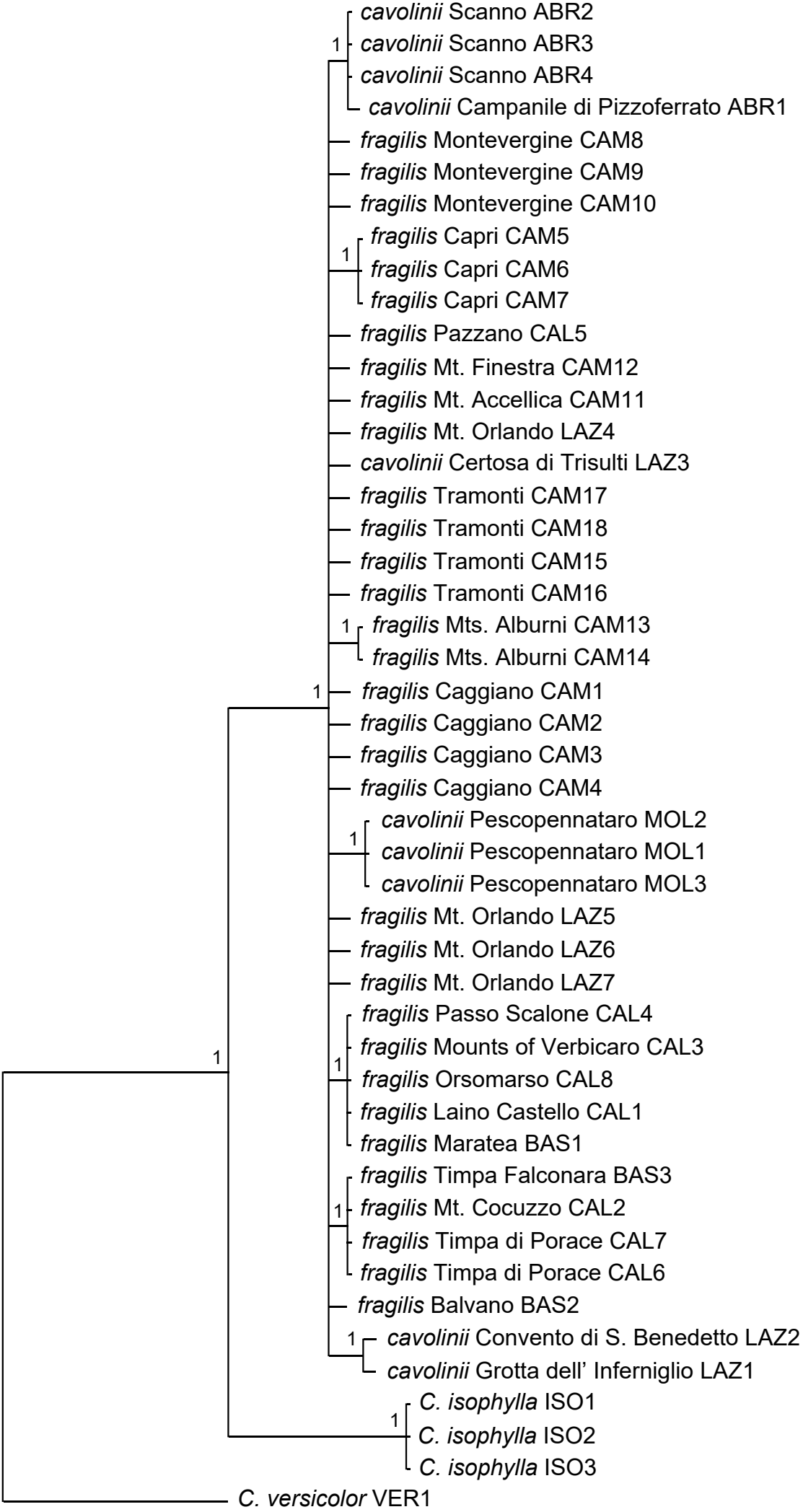

Supplement: Supplementary file 1 [file plants-13-03169-s001.zip › Figure S2.pdf]
